# KRAS-Driven Lung Adenocarcinoma and B Cell Infiltration: Novel Insights for Immunotherapy

**DOI:** 10.3390/cancers11081145

**Published:** 2019-08-09

**Authors:** Pinto Rosamaria, Petriella Daniela, Lacalamita Rosanna, Montrone Michele, Catino Annamaria, Pizzutilo Pamela, Botticella Maria Antonietta, Zito Francesco Alfredo, Del Bene Gabriella, Zonno Antonia, Tommasi Stefania, De Summa Simona

**Affiliations:** 1 Molecular Diagnostics and Pharmacogenetics Unit, IRCCS-Istituto Tumori “Giovanni Paolo II”, VialeOrazioFlacco 65, 70124 Bari (BA), Italy; 2 Medical Thoracic Oncology Unit, IRCCS-Istituto Tumori “Giovanni Paolo II”, VialeOrazioFlacco 65, 70124 Bari (BA), Italy; 3 Histopathological Unit, IRCCS-Istituto Tumori “Giovanni Paolo II”, VialeOrazioFlacco 65, 70124 Bari (BA), Italy; 4 Clinical Trial Center, IRCCS-Istituto Tumori “Giovanni Paolo II”, VialeOrazioFlacco 65, 70124 Bari (BA), Italy

**Keywords:** LUAD, immunotherapy, tumor microenvironment, B cells, *KRAS*

## Abstract

Non-small-cell lung cancer, histologically classified into adenocarcinoma (AD) and squamous cell carcinoma, is one of the most deadly malignancies worldwide. Lung AD (LUAD) could benefit of a plethora of target therapies and, in the last few years, also of immunotherapies. Here we focused on a real-life cohort of LUAD and The Cancer Genome Atlas (TCGA)-LUAD dataset aiming to gain insights into the immune contexture of such a malignancy. We explored the mutational status of 41 genes and the expression of 94 genes, related to immune-checkpoint, inflammation, and stromal microenvironment. Surprisingly, we found that our cohort has a very low mutational burden if we consider our panel as its surrogate. Regarding gene expression data, we identified 31 genes significantly deregulated in tumor tissues compared with a pool of normal samples. Unsupervised hierarchical clustering of the deregulated genes is able to identify two clusters of tumor samples, differently enriched in alterations in actionable genes. In particular, we identified a cluster enriched in patients carrying *KRAS* alterations. In silico deconvolution, that is the inferring of tumor microenvironment composition by gene expression data, through TIMER algorithm has been performed to explore immune microenvironment. Estimation performed on our gene expression matrix showed that B cell infiltration is lower in the *KRAS*-mutated enriched cluster, as in the TCGA-LUAD dataset. Such a finding has been validated in situ through immunohistochemistry in an independent cohort. Moreover, cases in LUAD-TCGA with low B cell infiltration have a significantly worse overall survival than those with higher levels. In the real-life cohort we observed that cases belonging to cluster enriched in *KRAS*-mutated patients have a poor outcome. LUAD driven by *KRAS* mutation represents an unmet clinical need, being refractory to pharmacological inhibition. Our results link *KRAS* mutations to B cell infiltration. Thus, the present findings could be helpful in a better definition of immunotherapeutic approaches for *KRAS* mutated patients.

## 1. Introduction

Lung cancer remains one of the most deadly malignancies worldwide. The majority of patients have non-small-cell lung cancer (NSCLC) and in an advanced stage [1]. They can be histologically classified into lung adenocarcinoma (LUAD) and squamous cell carcinoma, with the former being the most common subtype, comprising more than 50% of all NSCLC [1].

Historically, these patients were treated with platinum chemotherapy regardless of histological subtype. Despite an improvement in overall survival (OS) when compared with best supportive care, a therapeutic plateau has been reached with a response rate of about 20% and a median survival of 8–10 months [2]. Actionable genetic alterations (e.g., *EGFR* (growth factor receptor) mutations, ALK/ROS1 translocations) made it possible to treat patients in a targeted way, even if only a small percentage of them benefit from these approaches. In a different percentage of LUAD (20%–25%) the discovery of *KRAS* mutations has been associated with poorer overall survival (OS) than with *KRAS* wild-type tumors, particularly in the advanced-stage setting [3,4].

In order to improve the clinical outcome of these patients, a novel emerging approach is based on the immunotherapy that acts targeting specific immune regulatory checkpoints. In particular, Cytotoxic T-Lymphocyte Antigen 4, CTLA-4, and Programmed cell death protein 1/Programmed death-ligand1, PD-1/PD-L1, are the main checkpoints to be targeted [5]. Briefly, CTLA4 is involved in the early phase by reducing T-cell response to self-antigens; the axis PD-1/PD-L1 acts in the active stage of T-cell response in tumor microenvironment. Thus, targeting them lead to reduction of tumor growth through increasing the immune response. Biomarkers able to discriminate patients which benefits from immunotherapies are still under investigation. Tumor mutational burden, neoantigens, and PD-L1 expression are not sufficient probably because a deeper knowledge of tumor microenvironment (TME) is not taken into consideration. In particular, TME changes and the relationship with the biology of cancer cells (e.g., mutational status of peculiar genes or altered pathways) are needed. Indeed, conflicting results on the correlation among *EGFR/KRAS* (epidermal growth factor receptor/Kirsten Rat sarcoma virus) mutations and immune checkpoint inhibitors response have been evidenced. *KRAS*, which is considered as undruggable, has been demonstrated by several groups to be able to modulate the immune response, as in colorectal and pancreatic cancers [6,7,8].

Thus, to study how the mutational status of cancer cells could contribute to the enhancement of immunogenicity, should be able to indicate the sensitivity to immune checkpoint blockade [9]. It is well known that the interplay between immune microenvironment and cancer cells is highly dynamic in the different steps of cancer onset. Generally speaking, three important phases (elimination, equilibrium, escape) could resume the deep changes of the immune system in relation to carcinogenesis [10]. Such a process is called “immunoediting” and it is recognized as one of the hallmarks of cancer. Briefly, starting from the initial phases of immune surveillance and quiescence, respectively elimination and equilibrium, the immune system does not attack the tumor allowing its proliferation [11,12]. Several studies pointed out the different composition of TME of lung cancer, recently reviewed by Altorki et al. [13]. In the last few years, computational methods were developed to infer TME composition from bulk genomic data. These approaches, such as CIBERSORT [14] or TIMER [15] are deeply used to date to gain knowledge of TME.

In this study we explored the biological heterogeneity of the immune landscape of a real-life cohort of 20 patients affected by LUAD and of a cohort affected by LUAD and reported as TGCA dataset with the aim to identify potential biomarkers useful for a possible immune-therapy approach in these patients.

## 2. Results

### 2.1. Immune-Mutational Status

In the present study, we analyzed a cohort including 20 LUAD patients. The series comprises patients affected by advanced stage LUAD (stage IIIB-IV), carrying 7/20 (35%), 8/20 (40%), 8/20 (40%) *EGFR, KRAS*, and *TP53* alterations, respectively.

**Figure 1:**
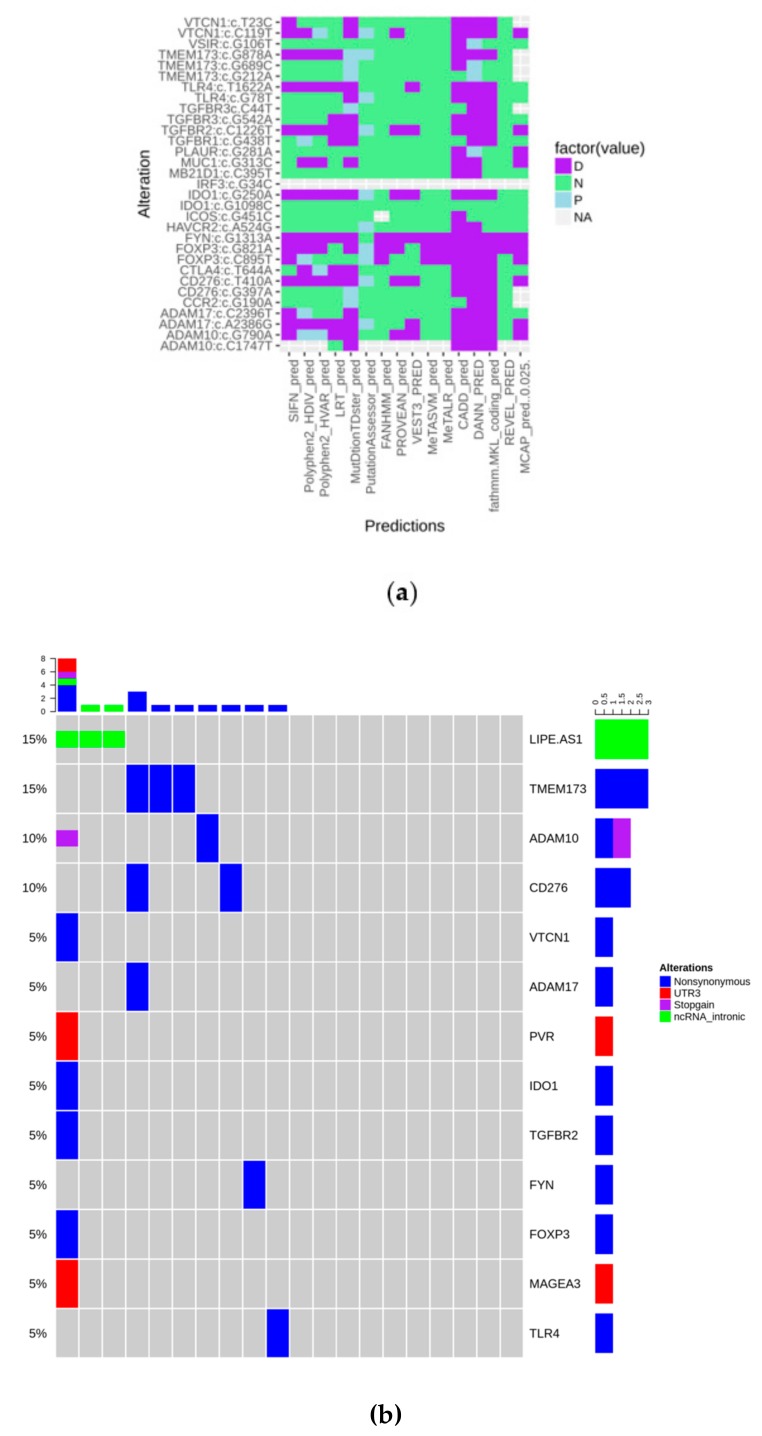
(**a**) Heatmap showing the predicted biological impact of detected alterations (D: Deleterious; N: Neutral; P: Predicted deleterious); (**b**) Oncoprint of the mutations that “survived” all the filtering steps.

To explore the mutational status of genes involved in immunological/inflammatory response, we designed a targeted sequencing panel including 41 genes. After filtering, as described in the Materials and Methods section, variants were annotated and their deleteriousness was predicted through 16 tools, as shown in Figure 1a. They were considered as pathogenic when at least 9/16 algorithms agreed in their prediction. Alterations, that “survived” the filtering steps included, regards 13 genes over 41. PVR c.*27G > A, MAGEA3 c.*41C > T, and LIPE-AS1 were retained because of their probable regulating role.

The oncoprint plot in Figure 1b shows that alterations have no peculiar distribution in the analyzed cohort, indicating that the “immune mutational status” is substantially wild-type. Moreover, considering our panel as a surrogate to measure tumor mutational burden, it could be observed that it is very low in the present series.

### 2.2. Immune Gene Expression Results and Integration with Overall Molecular Data

Immune gene expression evaluation was performed through a custom designed *targeted panel including 95 genes*. To explore deregulation, we compared our tumor samples with nonpathological samples through the DeSeq2 algorithm. We detected 31/95 genes significantly deregulated (Table 1). Gene expression results were confirmed on LUAD-TCGA dataset (*n* = 574) through the web-browser of TIMER (Supplementary Material Figure S1). Only *ITGAE, IL7*, and *HMGB1* expression observed in LUAD-TCGA have an opposite behavior with respect to what was found in our cohort.

**Figure 2:**
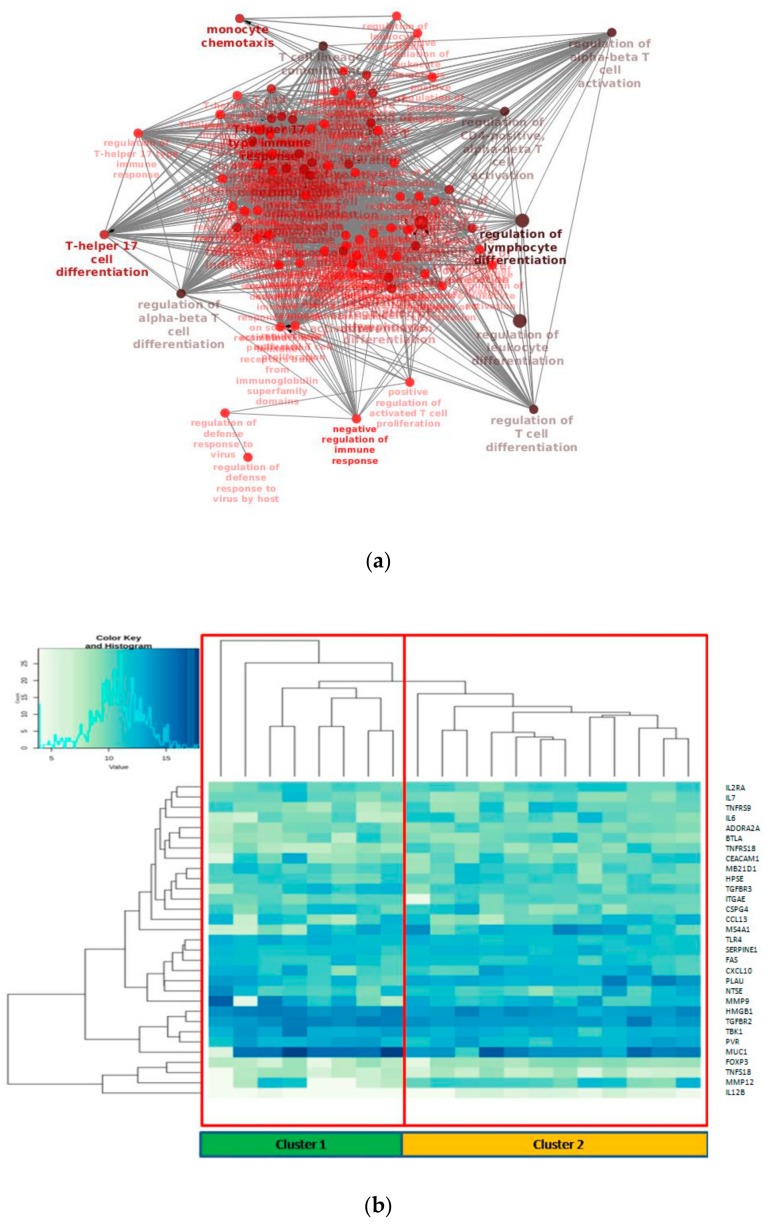
(**a**) Biological network of the enriched GO (Gene Ontology) terms related to differentially expressed genes in the tumor/normal setting; (**b**) Unsupervised hierarchical clustering of the 31 deregulated genes.

GO/KEGG enrichment of significantly deregulated genes has been depicted through Cluepedia/Cluego network (Figure 2a). In particular, it could be observed the enrichment of terms related to T cell differentiation, as could be expected, but more interestingly terms regarding T helper 17 lymphocytes (Th17) were found.

**Figure 3:**
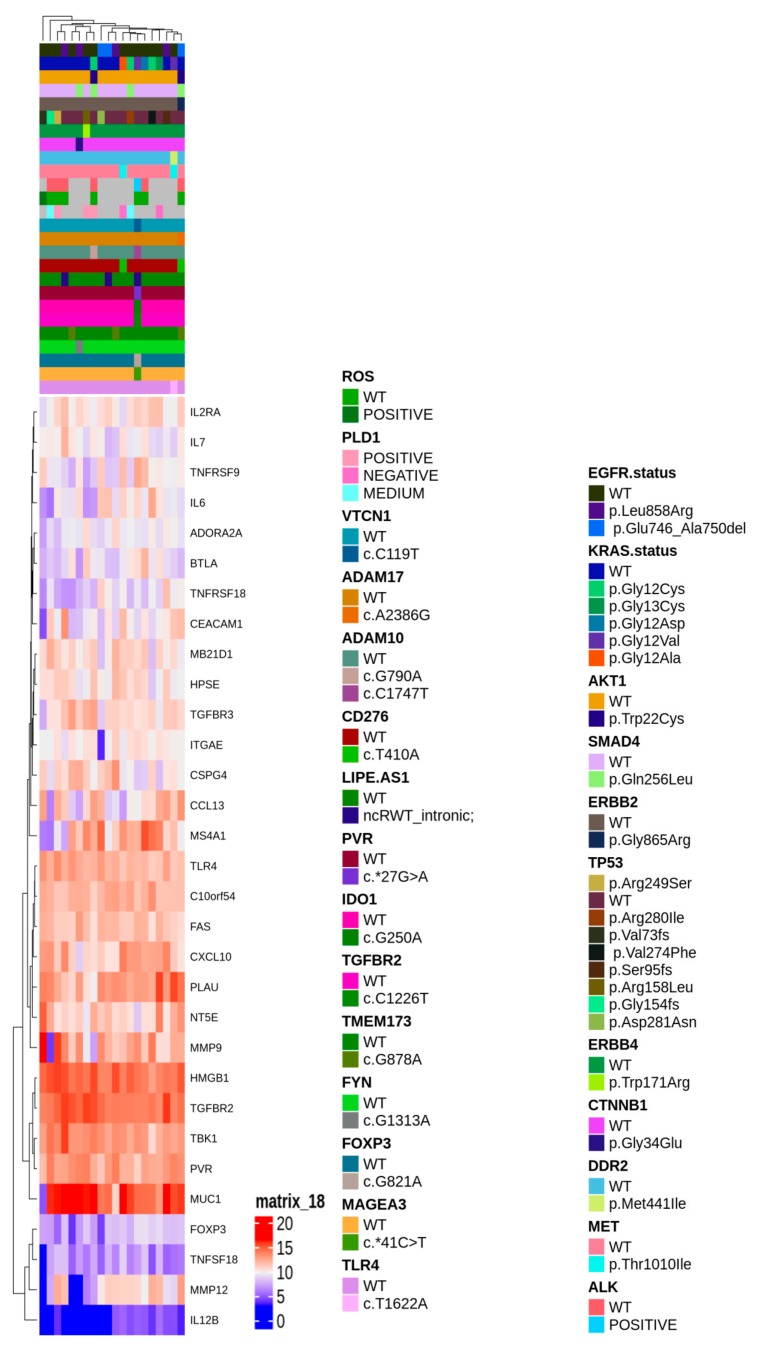

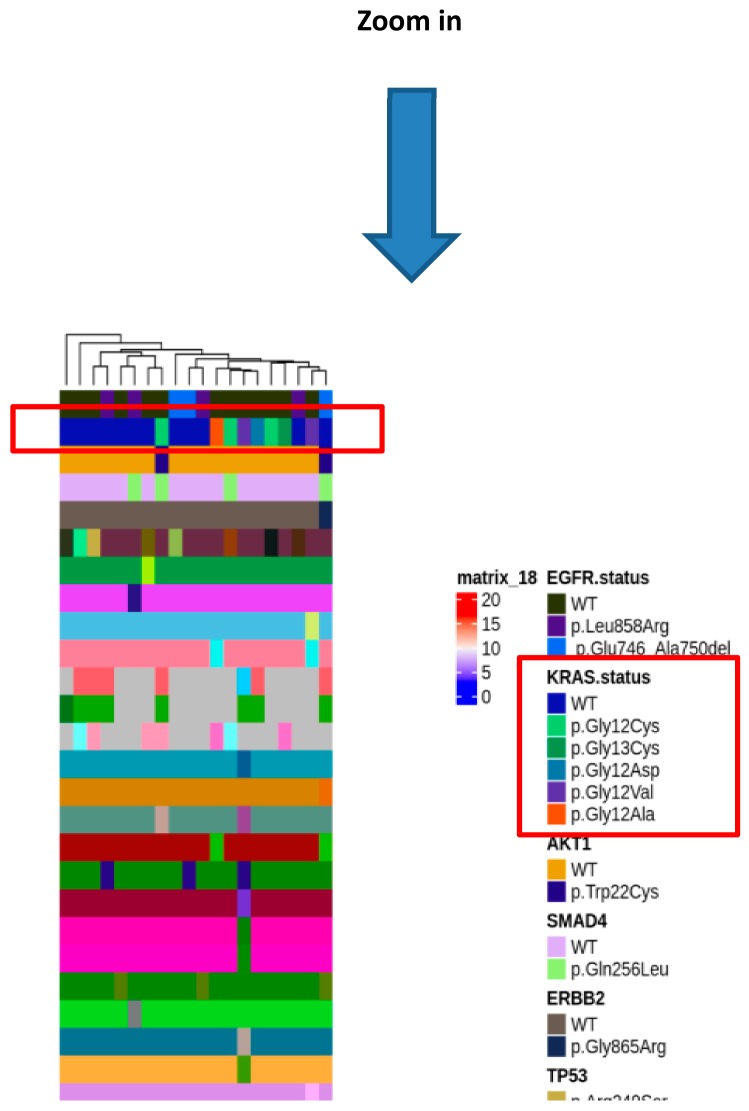
Complex heatmap annotated with all the genetic alterations detected in the *real-life cohort.*

To gain insight of such deregulated genes, unsupervised hierarchical clustering of LUAD tumor samples was performed, which splitted the cohort into 2 clusters (Figure 2b). Thus, we aimed to identify a molecular feature able to distinguish the two identified clusters. A complex heatmap (Figure 3) including all molecular data available for our cohort (colon and lung panel results, *ALK/ROS1* translocations and PD-L1 staining when available) was drawn. It could be seen that almost all *KRAS* mutated patients are grouped in cluster 2 (7/8 mutated cases in cluster 2) (Figure 3). In particular, alterations in *KRAS* are p.G12X and p.G13X, where X stands for the mutated amino acid.

### 2.3. Immune Infiltration Estimation and Overall Survival

The identification of two clusters differently enriched in *KRAS* mutation carrying patients and the identification of pathway/GO terms of the deregulated genes are not sufficient to infer the composition of microenvironment.

**Figure 4:**
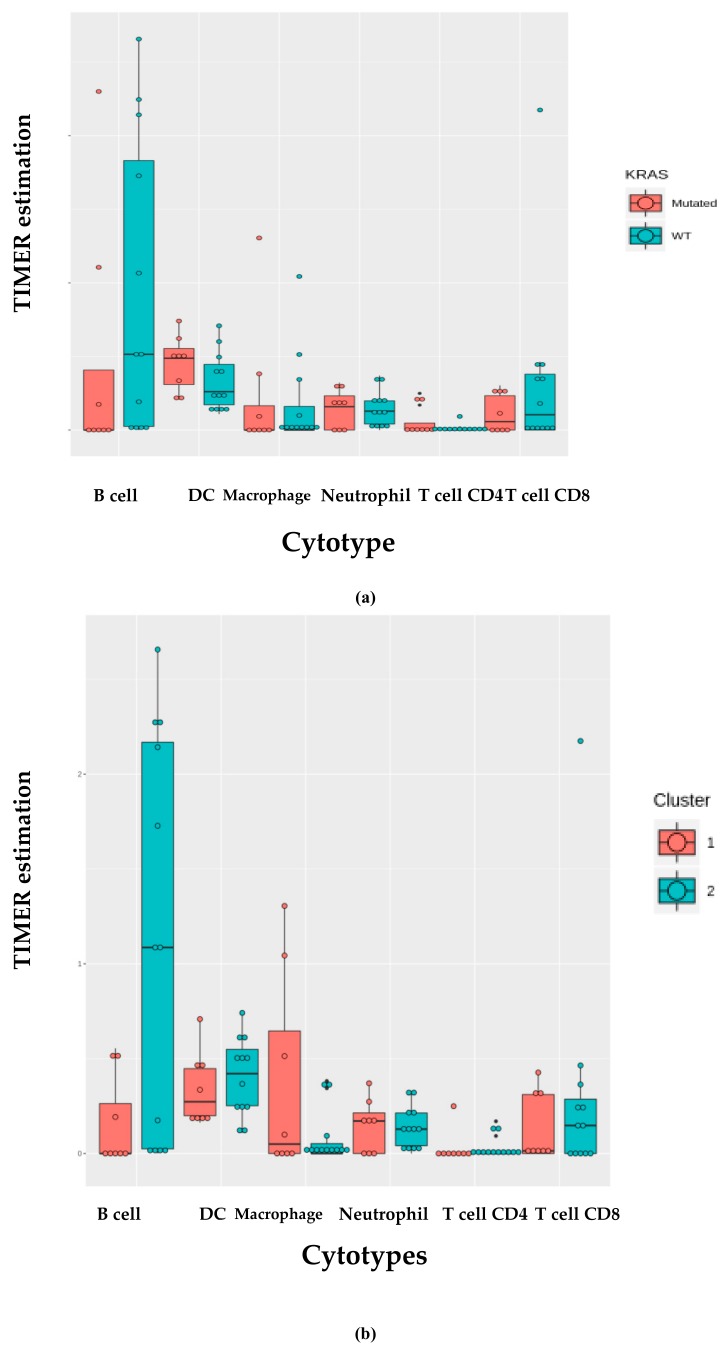

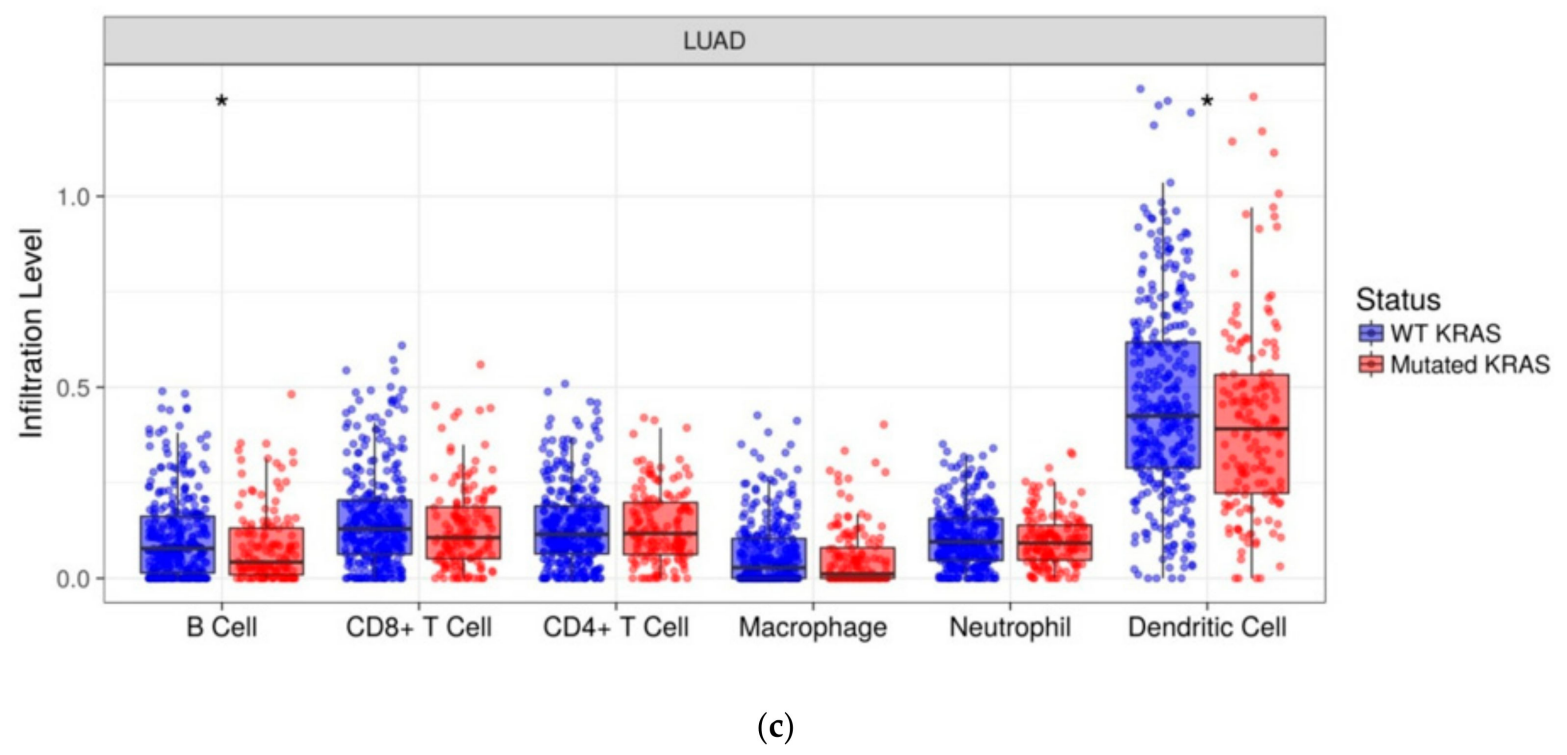
TIMER deconvolution from 95-gene expression matrix of real-life cohort stratifying for (**a**) *KRAS* mutational status and (**b**) cluster assignment; (**c**) TIMER estimation of TCGA-LUAD dataset.

We investigated the immune infiltration through TIMER deconvolution approach, which is more reliable than other methods (e.g., CIBERSORT that suffers from collinearity bias). Estimation performed on our gene expression matrix showed that, after stratification based both on cluster and *KRAS* mutational status, B cell infiltration is lower in both cluster 2 subsets and *KRAS* mutated patients than in cases belonging to cluster 1and in *KRAS* WT (*p* = 0.06) (Figure 4a,b). Notably, also in the LUAD-TCGA dataset, B cell infiltration is significantly low in *KRAS* mutated patients (Figure 4c). Dendritic cell infiltration, which is significantly lower in *KRAS* mutated cases than wild-type ones in LUAD-TCGA, has an opposite behavior in the real-life cohort we analyzed.

**Figure 5:**
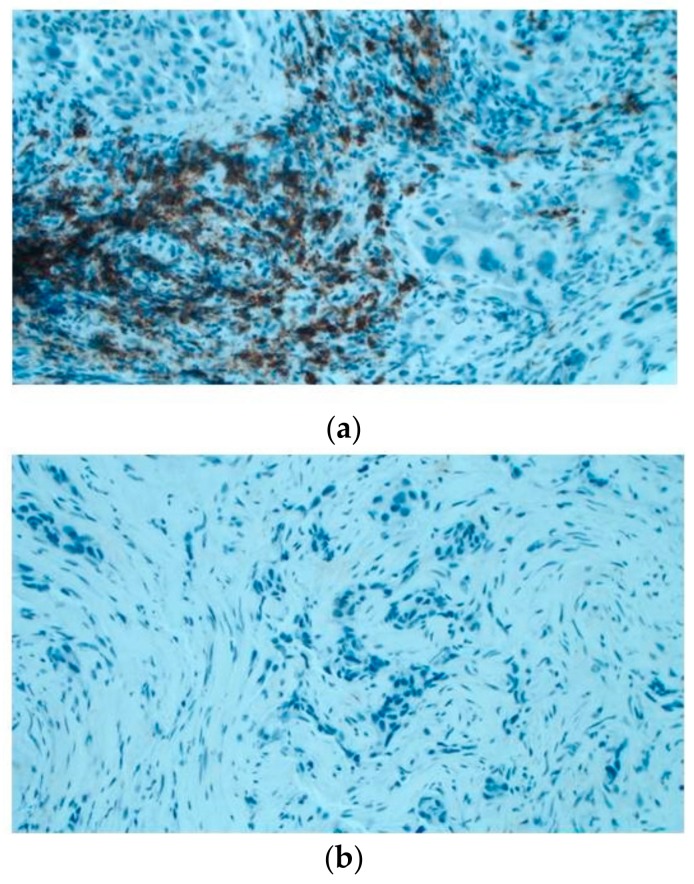
Immunoistochemical evaluation of B cell infiltration in (**a**) *KRAS* WT (60% of infiltration) and (**b**) mutated cases (0% infiltration), respectively (magnification: 20×) Moreover, cases in LUAD-TCGA with low B cell infiltration have a significantly worse overall survival than those with higher levels (Figure 6a). Interestingly, in the real-life cohort we observed the same results stratifying for presence/absence of *KRAS* mutation, with mutated patients having a poor outcome (Figure 6b). Moreover, mutated patients in cluster 2 have a worse overall survival than cluster 1 *KRAS* WT cases (Figure 6c).

Then, the amount of B lymphocyte infiltration has been evaluated in situ through immunohistochemistry in an independent cohort including 15 cases. It was observed that 5/6 (83%) of *KRAS* WT cases have B cell infiltration (20%–70% of total infiltration); whilst 5/9 *KRAS* (55%) mutated showed no B cell infiltration (Figure 5).

Of note, immune infiltration estimation on LUAD-TCGA data has been performed on RNA-Seq data whilst we performed estimation in our cohort using a matrix including 95 genes. Thus, our custom RNA panel has a good performance in TIMER estimation, being able to confirm the result related to B cell infiltration.

## 3. Discussion

The advent of immunotherapies posed a step forward in the treatment of many solid tumors, in particular LUAD. In the present study, both the analysis of real-life cohorts and TCGA-LUAD data allowed to gain insights into the biology of such a disease. Through the set-up of two NGS custom panels, both for DNA and RNA, including genes related to major immunity checkpoints and pathways, it was possible to dissect different aspects of LUAD. 41-gene DNA panel evidenced no mutational enrichment. This is an important finding because mutational status of genes involved in immunological/inflammatory pathways could be less important than their fine regulation. Tumor mutational burden (TMB) is a potential biomarker, also in NSCLC, for response to immunotherapy targeting PD-1/PD-L1 [16,17]. If our panel is considered as a surrogate of TMB, thus our result confirmed previous findings highlighting the lower mutational burden of LUAD with respect to squamous cell carcinoma [18,19]. Moreover, the role of TMB as a predictive biomarker is still controversial, both if it is considered solely or in combination with other ones, such as PD-L1 expression. Regarding 95-gene panel expression analysis, 31 genes were detected as significantly deregulated comparing tumor/normal samples. Unsupervised hierarchical clustering of tumor samples evidenced two clusters, which were found to be differently enriched in *KRAS*-mutant samples. *KRAS* mutations are detected almost exclusively in LUAD [20,21] and they are associated to poor survival, in particular in advanced stages [22,23]. Moreover, *KRAS* mutant is biologically heterogeneous in terms of type of alteration and concomitant mutations. The type of KRAS mutation has been dissected with respect to patient survival. No specific distribution has been observed of the nine types of mutations at codons 12, 13 e 61 in NSCLC in the three co-occurring mutation clusters [24]. However, in vitro studies highlighted different behaviors on the downstream pathways, such a PI3K-AKT affinity for *KRAS*-G12C or *KRAS*-G12V [25]. In NSCLC patients *KRAS*-G12C has been correlated to poorer overall survival than *KRAS*-G12D mutants or *KRAS*-G12A [26]. Nevertheless, the association of specific *KRAS* mutations to survival needs more data and appropriate study design. The cohort of the present study carries only alterations at codon 12 and 13. In our cohort, patients in *KRAS*-mutant enriched cluster, 33% of samples carry a mutation in *TP53*, but only the half of them are *KRAS/TP53* co-mutant. Of note, *TP53* mutants in the other cluster are all *KRAS* wild-type. Thus, cluster 2 is enriched both in *KRAS* mutation carriers and *KRAS*/TP53 double mutants. Skoulidis et al. identified three subgroups of *KRAS*-driven LUAD based on co-occurring mutations. In particular, the so-called KP cluster, including *KRAS*/*TP53* mutants, has been demonstrated to be featured by immunoediting, T-cell inflammation, and resistance to adaptive immune response and also JAK-STAT pathway activation and interferon signaling [24].

To gain a deeper knowledge of the microenvironment, TCGA-LUAD cohort has been analyzed through TIMER, a deconvolution algorithm, which highlighted a significant low infiltration level of B-cells and dentritic cells in *KRAS* mutants. B-cell result has been confirmed using the 95-gene panel expression matrix both stratifying by *KRAS* mutational status and clustering information and by IHC on an independent cohort. The function in anti-tumor immunity of B cells has emerged in lung cancer in several studies. Indeed, it has been shown that B lymphocytes are able to promote T cell activation and expansion. B cells, which are responsible for humoral immunity, are able in lung to differentiate into plasma cells producing antibodies against tumor antigens (e.g., TP53, NY-ESO-1) and the high concentration of tumor-antigen antibodies has been associated to a high proportion of follicular B cells [27]. Plasma cells, follicular B cells [28], and co-localization of CD8+ and CD4+ T cells and B lymphocytes are associated to long overall survival in NSCLC patients [29,30]. Such a result has been confirmed also in the TCGA-LUAD dataset. However, the association of B cell infiltration and *KRAS* alteration is the most interesting finding of the present study. The role of *KRAS* in the modulation of the tumor microenvironment has been demonstrated not only for the immune component but also the stromal one. GO/KEGG analysis evidenced a significant enrichment of terms related to Th17 lymphocytes and in the *KRAS*-mutants enriched cluster the upregulation of IL6 and MMP12 has been detected, confirming the crosstalk between tumor and Th17 cells [31,32]. To date, few data have demonstrated the link between the mutational status of *KRAS* and B cell. The role of *KRAS* in the B lymphopoiesis has been shown by Chen et al. [33] that demonstrated how the lack of *KRAS* in murine model impairs B cell development and maturation. Nevertheless, models to study the crosstalk between *KRAS* mutant cells and B lymphocytes are mandatory because this relationship needs to be studied to improve the (immune)therapeutic setting of LUAD patients. Indeed, recently Meng et al. [34] demonstrated the ability to target tumor neoantigens, as *KRAS* mutants, eliciting the humoral immune response through infiltrating B cells in pancreatic cancer. Such a mechanism is promising because it is similar to the NY-ESO-1 approach in melanoma [35,36]. Regarding NSCLC, it has been shown that in vitro cultivated infiltrating B cells can present antigens and activate CD4+ T cells [37]. It is a very interesting study on the role of immune regulation in TME of NSCLC. However, a major effort is needed to link mutations harbored by tumor to the capacity to present antigens of B cells.

In particular, *KRAS* mutation/low B cell infiltration association with respect to overall survival highlighted the possibility to set-up novel immunotherapies, such as the B lymphocytes-based approach. Indeed, recent clinical studies testing allogeneic B cells from PBMCs (peripheral blood mononuclear cells) of donors in renal cell tumor and melanoma [38] and adoptive B-cell transfer approach [39] showed promising results.

In conclusion, the present study, exploiting through genomic data the biology of LUAD, confirmed the importance of the mutational status regarding the relationship between the tumor cell and microenvironment. Intriguingly, the association of KRAS alterations to B cell infiltration and survival shed a light to the set-up of new therapeutic approaches. Moreover, the results coming from deconvolution on the expression matrix of the custom targeted panel demonstrated that it could be a useful tool to explore the TME composition, as demonstrated through the TCGA-LUAD dataset molecular data, and in situ validations.

## 4. Materials and Methods

### 4.1. Sample Cohort

Twenty advanced-stage (IIIB and IV) NSCLC patients were enrolled by the Department of Thoracic Oncology of the Giovanni Paolo II Institute of Bari between November 2016 and May 2017.Evaluation of survival was performed according to RECIST 1.1 criteria with a median time OS time of 9.67 months. Moreover 8 patients who underwent surgery with lung tumor free diagnosis were enrolled by the Department of Surgery of San Paolo Hospital, Bari. All cases were diagnosed according to the current WHO guidelines. A cohort including 15 LUAD cases, whose *KRAS* mutational status was known, was used to validate deconvolution results by the in situ approach.

The study was approved by the local Ethics Committee of the IRCCS “Giovanni Paolo II” of Bari (CE prot. n. 540/2015) and was performed in accordance with the international standards of good clinical practice. All patients signed informed consent.

Blood samples from healthy patients were collected and stored as germline biological material.

### 4.2. DNA and RNA Extraction

Between three to six FFPE tissue sections (6 μm thick) with adequate tumor cellularity selected by a pathologist (>50%), were macro dissected and subjected to DNA and RNA isolation using QIAamp DNA FFPE Tissue Kit (Qiagen, Hilden, Germany) and RNeasy FFPE Kit(Qiagen), respectively, according to manufacturers’ protocols. DNA was also isolated from blood samples using QIAamp DNA Blood Midi Kit (Qiagen). DNA and RNA concentration was determined using Qubit 2.0 fluorometer (Thermo Fisher Scientific, Waltham, MA, USA).

### 4.3. Ion Torrent PGM Sequencing

Sequencing was performed as previously reported in [40]. Briefly, two custom panels were designed through the Ion Ampliseq designer tool, one including the coding region of 41 genes to detect DNA alterations and one to study the gene expression of 95 genes. Both of them include genes involved in immune regulation and inflammation. Moreover, also CE-IVD Oncomine Solid Tumor (Thermo Fischer Scientific) was used to assess the mutational status of actionable genes.

All generated reads were aligned to human genome hg19 using the Torrent Suite Server.

### 4.4. Variant Calling and Filtering

Variant calling and filtering was described in [40]. Briefly, the callsetwas generated merging results from the Somatic High-Stringency Variant Caller plugin of Torrent Suite and Vardict [41] algorithm. Germline variants were filtered out using a pool of healthy controls. Annovar [42] was used to functionally annotate variants. Regarding results of the CE-IVD Oncomine Solid Tumor panel, an optimized Ion Reporter workflow was used and only variants annotated in COSMIC (Catalog Of Somatic Mutations in Cancer) database (https://cancer.sanger.ac.uk/cosmic).

The OncoPrint plot was designed with the ComplexHeatmap R package [43].

### 4.5. Gene Expression Detection

Gene expression was evaluated, as stated above, through a custom panel including 95 genes. Ten (10)ng of input RNA were reverse transcribed for target enrichment by the Ion AmpliSeq Library Kit 2.0 (Thermo Fisher Scientific). Libraries were then quantified and pooled in an equimolar ratio. Then libraries were pooled and templated using the Ion OneTouch 2 system. Sequencing of the Ion 318 chip was carried out on the Personal Genome Machine (PGM, Thermo Fisher Scientific) according to the Ion PGM Sequencing 200 Kit (Thermo Fisher Scientific). Read count matrix was downloaded from the Torrent Server and analyzed with DeSeq2 R package [44]. Deconvolution was performed through the web interface of TIMER (https://cistrome.shinyapps.io/timer/) both to the analyzed TCGA-LUAD dataset and real-life cohort [10].

### 4.6. Immunohistochemistry and Immunohistochemical Assessment

Paraffin-embedded tissue sections of patients fixed in formalin were cut into 4µm, the slides were processed and stained by the indirect immunoperoxidase method using the Dako Omnis Platform (Dako, Santa Clara, CA, USA). The slides underwent deparaffinization, followed by antigen retrieval with EnVision FLEX Target Retrieval Solution, Low pH for PD-L1, and High pH for CD20. The sections were incubated with primary antibodies Monoclonal Mouse Anti-Human PD-L1 (Clone 22C3; Dako Omnis; diluited at 1:30) and Monoclonal Mouse Anti-Human CD20cy (Clone L26; Dako Omnis; ready-to-Use). After endogenous peroxidase activity blocking with EnVision FLEX Peroxidase-Blocking Reagent, the products of the antigen–antibody reactions were visualized by EnVision FLEX DAB + Chromogen and EnVsion FLEX Substrate Buffer. Cell nuclei were stained with Hematoxylin (8 min). Tonsil FFPE sections were used as positive controls. For negative control, the primary antibody was omitted.

## 5. Conclusions

The present paper, for the first time, links *KRAS* mutational status, which is responsible for a poor survival in LUAD patients, and B cell infiltration, which, in turn, has been indicated as a good prognostic marker. Notwithstanding the small size of the real-life cohort, the in silico exploration of the TCGA-LUAD dataset and the immunohistochemistry validation of B cell infiltration on an independent cohort indicate that the results could be further explored in a more extensive way. The aforementioned relationship between *KRAS* and B lymphocytes, moreover, will be explored in a larger cohort, including also LUAD patients treated with standard immunotherapies. To gain functional insights into the response to immune checkpoint inhibitors and the link between *KRAS* and B cells,anin vistrostudy will be planned.

## Figures and Tables

**Figure 6:**
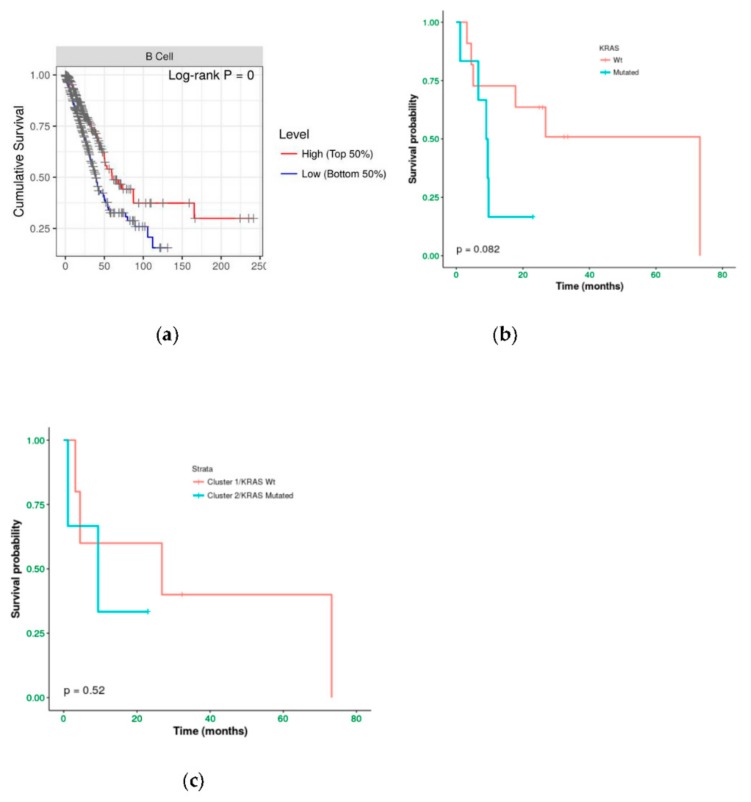
Kaplan–Meier curves of (**a**) TCGA-LUAD cohort stratifying for B cell infiltration and real-life cohort stratified for (**b**) *KRAS* mutational status and (**c**) cluster assignment.

**Table 1:** Significantly deregulated genes in the tumor/normal comparison.

	log2FoldChange	Adjusted *p*-Value
C10orf54	−1.63	8.06 × 10^−8^
TGFBR3	−2.46	1.98 × 10^−7^
IL6	−3.39	2.43 × 10^−7^
PLAU	2.83	3.85 × 10^−7^
MMP12	5.63	5.84 × 10^−5^
TNFRSF9	2.12	0.0002
ADORA2A	−1.38	0.0003
IL2RA	1.32	0.0011
FOXP3	2.75	0.0013
NT5E	2.64	0.0013
MUC1	2.48	0.0015
TNFRSF18	2.05	0.0039
TNFSF18	2.87	0.0039
MMP9	2.17	0.0045
HPSE	1.26	0.0051
IL12B	5.57	0.0051
CSPG4	−1.59	0.0079
CEACAM1	1.75	0.010
TGFBR2	−0.89	0.010
ITGAE	1.21	0.014
BTLA	1.61	0.018
TLR4	−0.89	0.026
TBK1	0.98	0.026
IL7	0.88	0.028
FAS	−0.73	0.029
CXCL10	1.19	0.034
MS4A1	1.79	0.034
HMGB1	0.72	0.040
MB21D1	0.97	0.042
SERPINE1	−1.23	0.042
CCL13	1.72	0.048

## Supplementary Materials

The following are available online at https://www.mdpi.com/2072-6694/11/8/1145/s1, Figure S1: Expression of the deregulated genes in the real life cohort in TCGA-LUAD dataset.
