# Supplementary material for: KRAS-Driven Lung Adenocarcinoma and B Cell Infiltration: Novel Insights for Immunotherapy
Source: Cancers (Basel). 2019 Aug 9;11(8):1145. doi: 10.3390/cancers11081145 (PMC6721568; doi:10.3390/cancers11081145)
Supplement: Supplementary file 1 [file cancers-11-01145-s001.pdf]

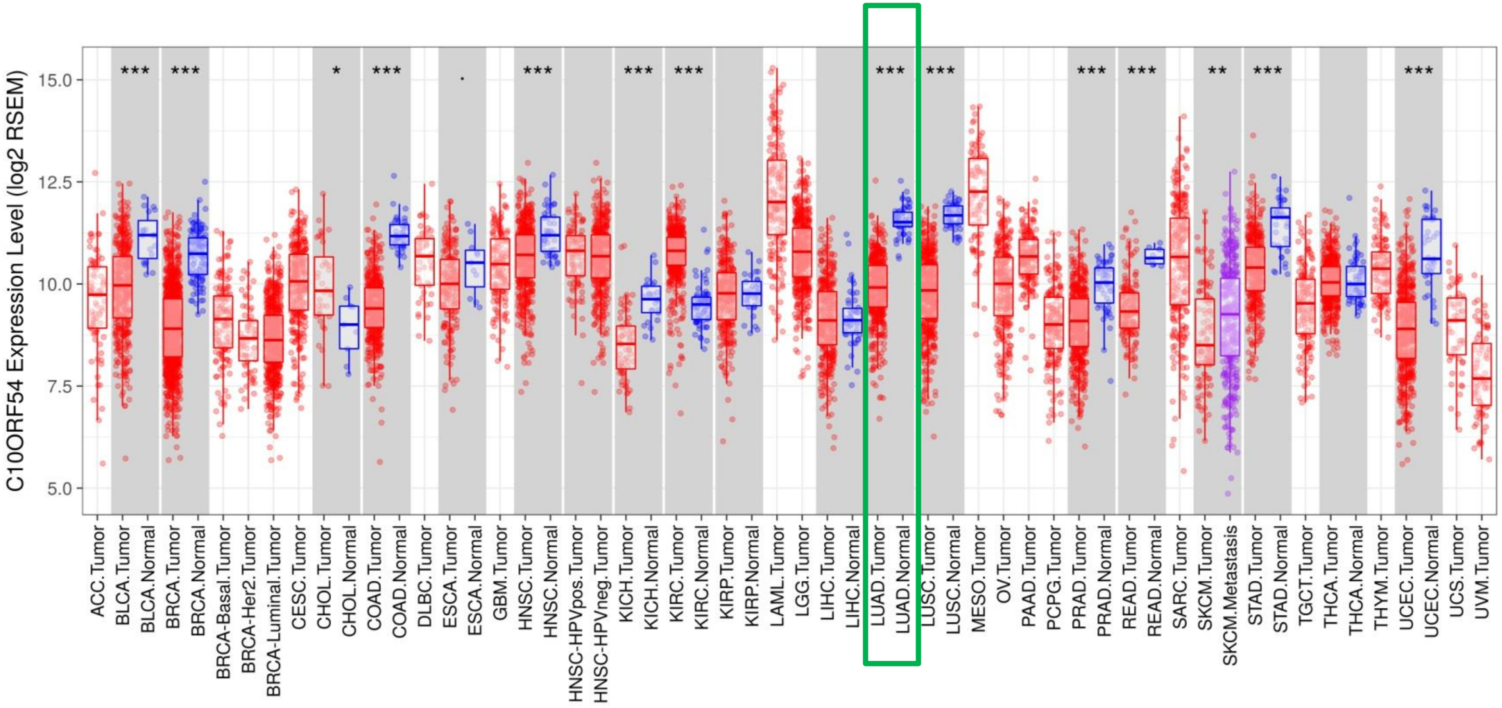

C10ORF54 Expression Level (log2 RSEM)
15.0
12.5
10.0
7.5
5.0
***
***
*
***
.
***
***
***
***
***
***
***
**
***
***
ACC.Tumor
BLCA.Tumor
BLCA.Normal
BRCA.Tumor
BRCA.Normal
BRCA-Basal.Tumor
BRCA-Her2.Tumor
BRCA-Luminal.Tumor
CESC.Tumor
CHOL.Tumor
CHOL.Normal
COAD.Tumor
COAD.Normal
DLBC.Tumor
ESCA.Tumor
ESCA.Normal
GBM.Tumor
HNSC.Tumor
HNSC.Normal
HNSC-HPVpos.Tumor
HNSC-HPVneg.Tumor
KICH.Tumor
KICH.Normal
KIRC.Tumor
KIRC.Normal
KIRP.Tumor
KIRP.Normal
LAML.Tumor
LGG.Tumor
LIHC.Tumor
LIHC.Normal
LUAD.Tumor
LUAD.Normal
LUSC.Tumor
LUSC.Normal
MESO.Tumor
OV.Tumor
PAAD.Tumor
PCPG.Tumor
PRAD.Tumor
PRAD.Normal
READ.Tumor
READ.Normal
SARC.Tumor
SKCM.Tumor
SKCM.Metastasis
STAD.Tumor
STAD.Normal
TGCT.Tumor
THCA.Tumor
THCA.Normal
THYM.Tumor
UCEC.Tumor
UCEC.Normal
UCS.Tumor
UVM.Tumor

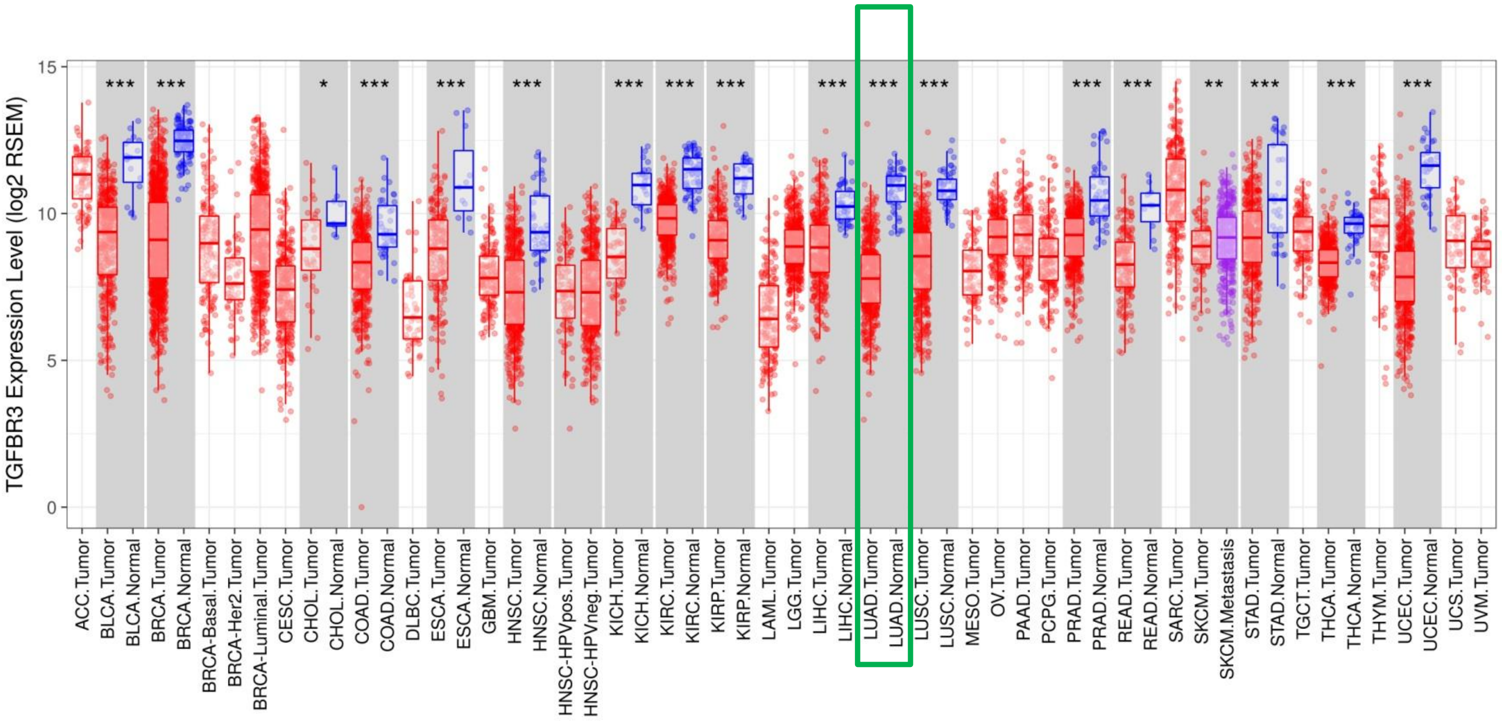
TGFBR3 Expression Level (log2 RSEM)
15
10
5
0
***
***
*
***
***
***
***
***
***
***
***
***
***
***
**
***
***
***
ACC.Tumor
BLCA.Tumor
BLCA.Normal
BRCA.Tumor
BRCA.Normal
BRCA-Basal.Tumor
BRCA-Her2.Tumor
BRCA-Luminal.Tumor
CESC.Tumor
CHOL.Tumor
CHOL.Normal
COAD.Tumor
COAD.Normal
DLBC.Tumor
ESCA.Tumor
ESCA.Normal
GBM.Tumor
HNSC.Tumor
HNSC.Normal
HNSC-HPVpos.Tumor
HNSC-HPVneg.Tumor
KICH.Tumor
KICH.Normal
KIRC.Tumor
KIRC.Normal
KIRP.Tumor
KIRP.Normal
LAML.Tumor
LGG.Tumor
LIHC.Tumor
LIHC.Normal
LUAD.Tumor
LUAD.Normal
LUSC.Tumor
LUSC.Normal
MESO.Tumor
OV.Tumor
PAAD.Tumor
PCPG.Tumor
PRAD.Tumor
PRAD.Normal
READ.Tumor
READ.Normal
SARC.Tumor
SKCM.Tumor
SKCM.Metastasis
STAD.Tumor
STAD.Normal
TGCT.Tumor
THCA.Tumor
THCA.Normal
THYM.Tumor
UCEC.Tumor
UCEC.Normal
UCS.Tumor
UVM.Tumor

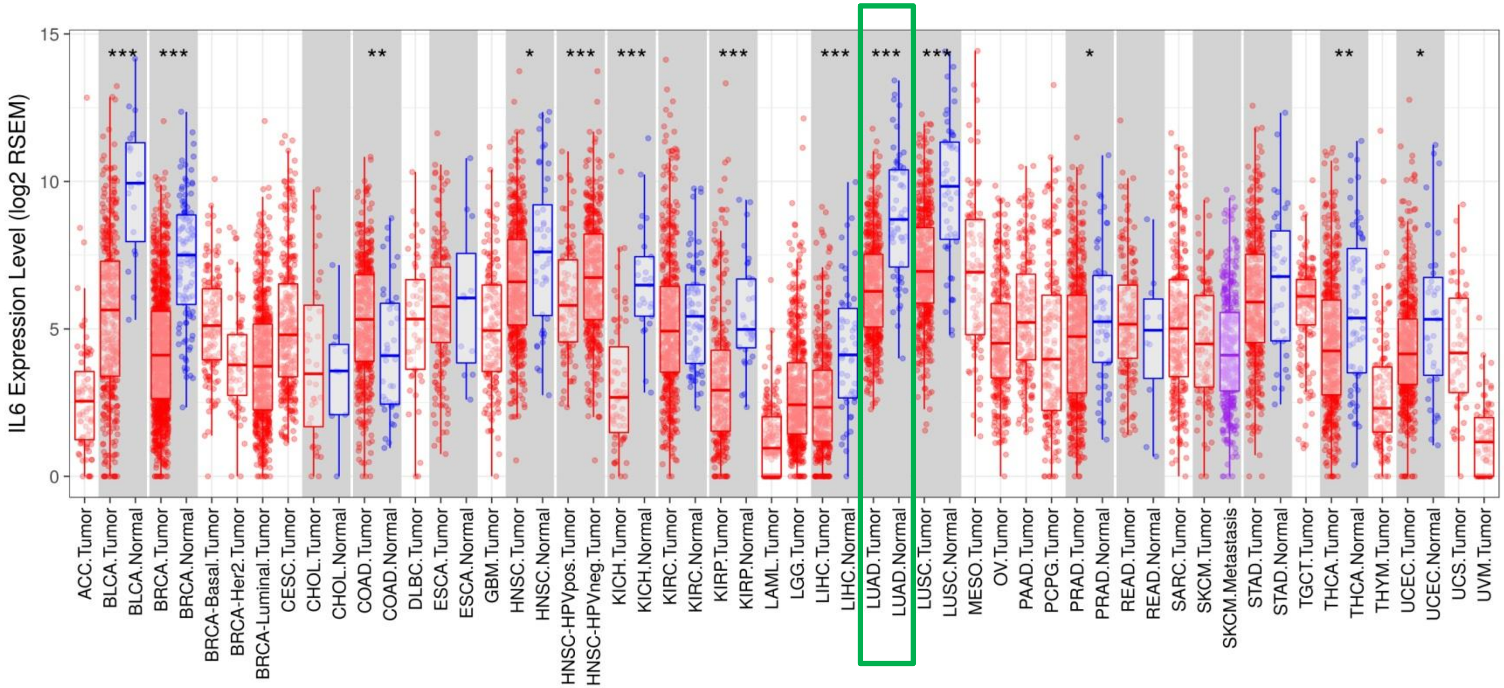

IL6 Expression Level (log2 RSEM)
0
5
10
15
*** *** ** * *** *** *** *** *** *** * ** *
ACC.Tumor
BLCA.Tumor
BLCA.Normal
BRCA.Tumor
BRCA.Normal
BRCA-Basal.Tumor
BRCA-Her2.Tumor
BRCA-Luminal.Tumor
CESC.Tumor
CHOL.Tumor
CHOL.Normal
COAD.Tumor
COAD.Normal
DLBC.Tumor
ESCA.Tumor
ESCA.Normal
GBM.Tumor
HNSC.Tumor
HNSC.Normal
HNSC-HPVpos.Tumor
HNSC-HPVneg.Tumor
KICH.Tumor
KICH.Normal
KIRC.Tumor
KIRC.Normal
KIRP.Tumor
KIRP.Normal
LAML.Tumor
LGG.Tumor
LIHC.Tumor
LIHC.Normal
LUAD.Tumor
LUAD.Normal
LUSC.Tumor
LUSC.Normal
MESO.Tumor
OV.Tumor
PAAD.Tumor
PCPG.Tumor
PRAD.Tumor
PRAD.Normal
READ.Tumor
READ.Normal
SARC.Tumor
SKCM.Tumor
SKCM.Metastasis
STAD.Tumor
STAD.Normal
TGCT.Tumor
THCA.Tumor
THCA.Normal
THYM.Tumor
UCEC.Tumor
UCEC.Normal
UCS.Tumor
UVM.Tumor

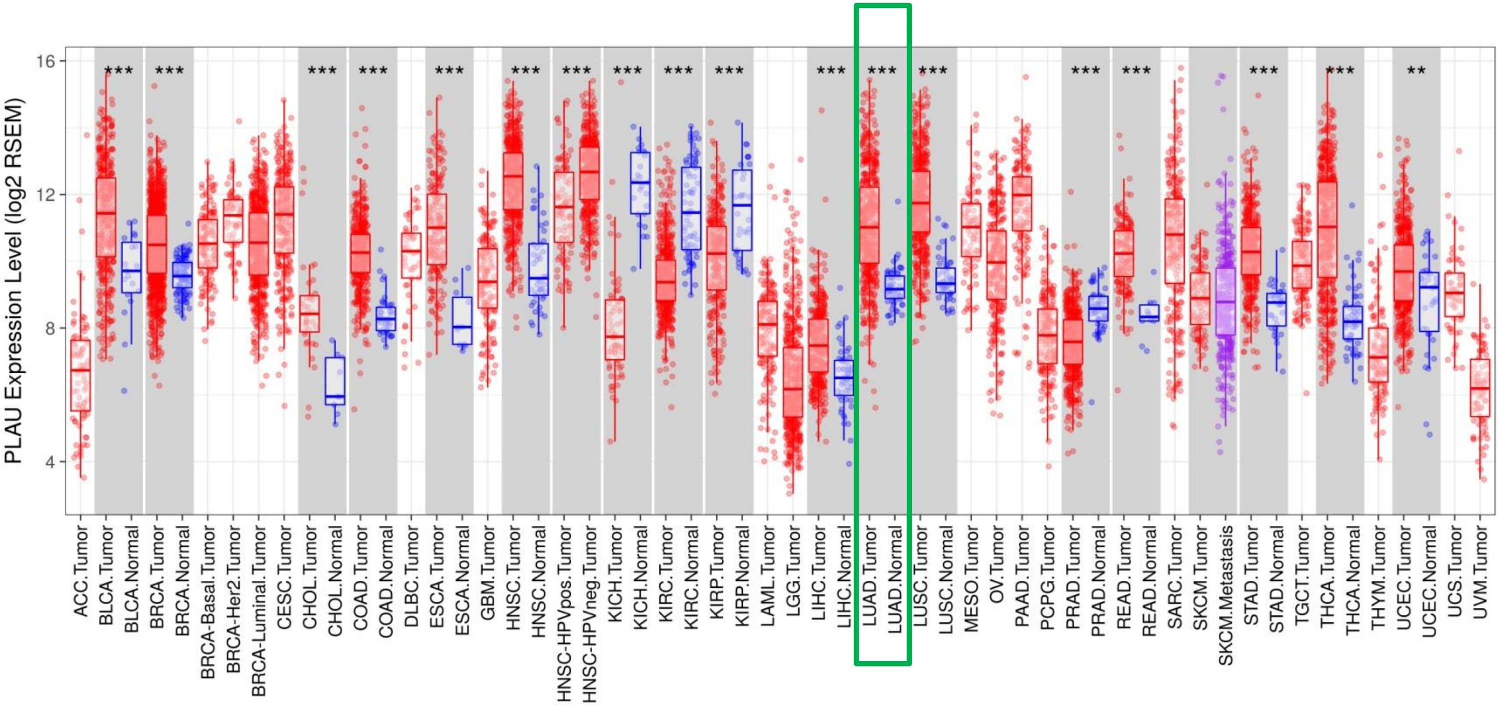
PLAU Expression Level (log2 RSEM)
16
12
8
4
***
**
ACC.Tumor
BLCA.Tumor
BLCA.Normal
BRCA.Tumor
BRCA.Normal
BRCA-Basal.Tumor
BRCA-Her2.Tumor
BRCA-Luminal.Tumor
CESC.Tumor
CHOL.Tumor
CHOL.Normal
COAD.Tumor
COAD.Normal
DLBC.Tumor
ESCA.Tumor
ESCA.Normal
GBM.Tumor
HNSC.Tumor
HNSC.Normal
HNSC-HPVpos.Tumor
HNSC-HPVneg.Tumor
KICH.Tumor
KICH.Normal
KIRC.Tumor
KIRC.Normal
KIRP.Tumor
KIRP.Normal
LAML.Tumor
LGG.Tumor
LIHC.Tumor
LIHC.Normal
LUAD.Tumor
LUAD.Normal
LUSC.Tumor
LUSC.Normal
MESO.Tumor
OV.Tumor
PAAD.Tumor
PCPG.Tumor
PRAD.Tumor
PRAD.Normal
READ.Tumor
READ.Normal
SARC.Tumor
SKCM.Tumor
SKCM.Metastasis
STAD.Tumor
STAD.Normal
TGCT.Tumor
THCA.Tumor
THCA.Normal
THYM.Tumor
UCEC.Tumor
UCEC.Normal
UCS.Tumor
UVM.Tumor

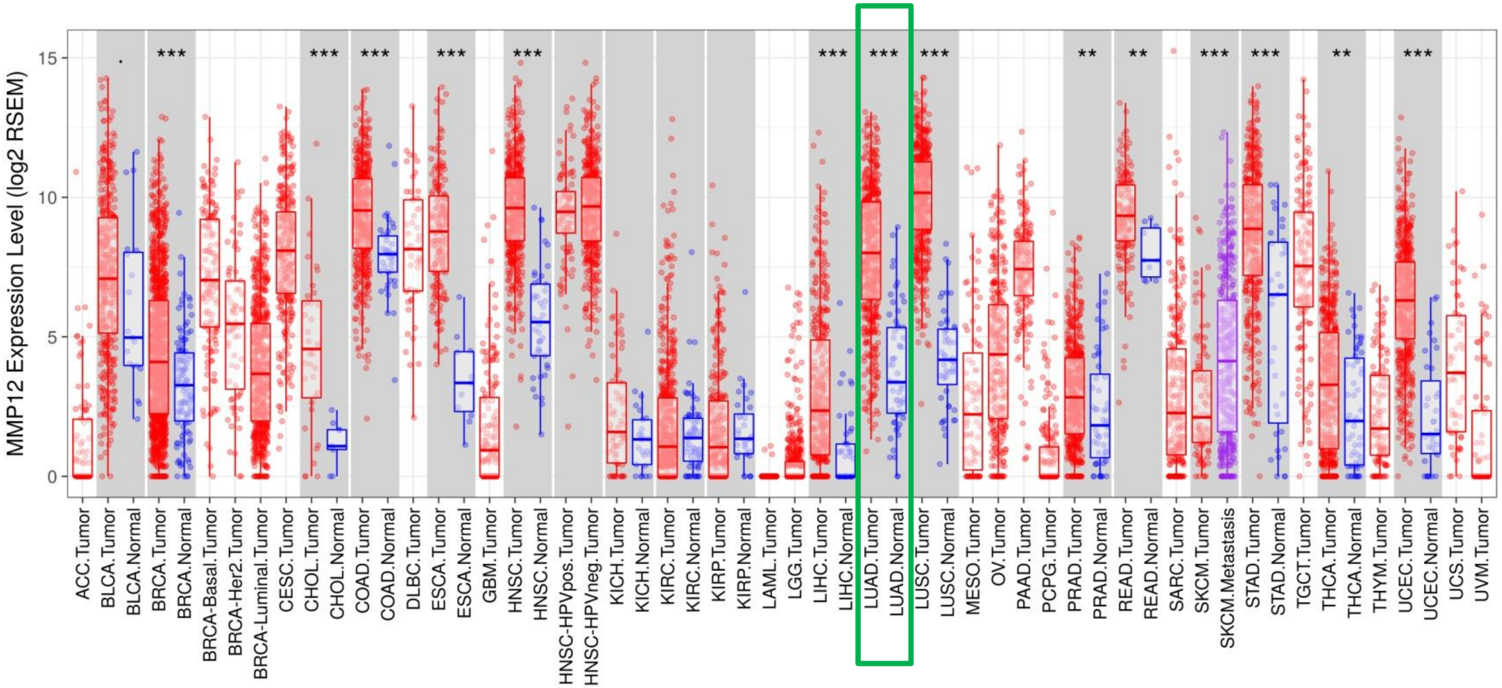
MMP12 Expression Level (log2 RSEM)
0
5
10
15
***
**
ACC.Tumor
BLCA.Tumor
BLCA.Normal
BRCA.Tumor
BRCA.Normal
BRCA-Basal.Tumor
BRCA-Her2.Tumor
BRCA-Luminal.Tumor
CESC.Tumor
CHOL.Tumor
CHOL.Normal
COAD.Tumor
COAD.Normal
DLBC.Tumor
ESCA.Tumor
ESCA.Normal
GBM.Tumor
HNSC.Tumor
HNSC.Normal
HNSC-HPVpos.Tumor
HNSC-HPVneg.Tumor
KICH.Tumor
KICH.Normal
KIRC.Tumor
KIRC.Normal
KIRP.Tumor
KIRP.Normal
LAML.Tumor
LGG.Tumor
LIHC.Tumor
LIHC.Normal
LUAD.Tumor
LUAD.Normal
LUSC.Tumor
LUSC.Normal
MESO.Tumor
OV.Tumor
PAAD.Tumor
PCPG.Tumor
PRAD.Tumor
PRAD.Normal
READ.Tumor
READ.Normal
SARC.Tumor
SKCM.Tumor
SKCM.Metastasis
STAD.Tumor
STAD.Normal
TGCT.Tumor
THCA.Tumor
THCA.Normal
THYM.Tumor
UCEC.Tumor
UCEC.Normal
UCS.Tumor
UVM.Tumor

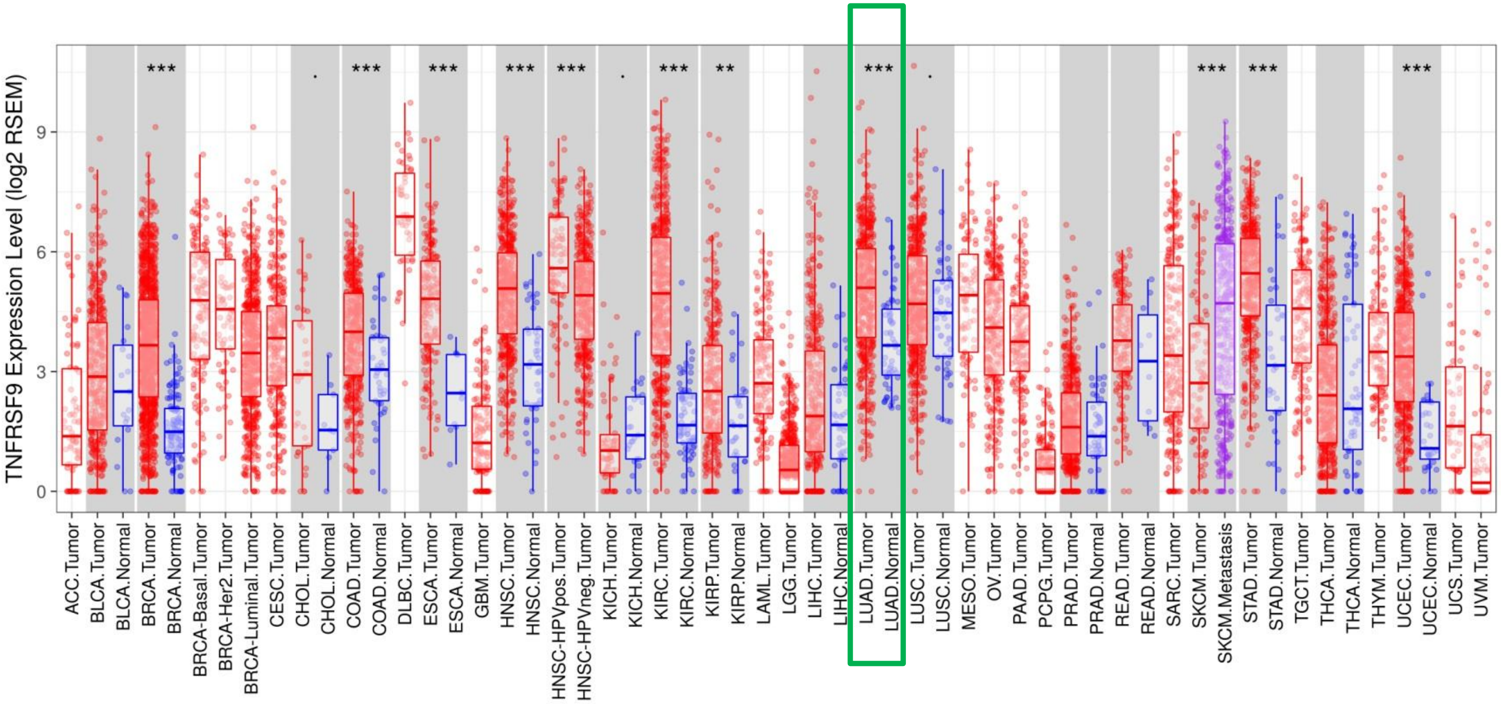

TNFRSF9 Expression Level (log2 RSEM)
0
3
6
9
***
.
***
***
***
***
.
***
**
***
.
***
***
***
ACC.Tumor
BLCA.Tumor
BLCA.Normal
BRCA.Tumor
BRCA.Normal
BRCA-Basal.Tumor
BRCA-Her2.Tumor
BRCA-Luminal.Tumor
CESC.Tumor
CHOL.Tumor
CHOL.Normal
COAD.Tumor
COAD.Normal
DLBC.Tumor
ESCA.Tumor
ESCA.Normal
GBM.Tumor
HNSC.Tumor
HNSC.Normal
HNSC-HPVpos.Tumor
HNSC-HPVneg.Tumor
KICH.Tumor
KICH.Normal
KIRC.Tumor
KIRC.Normal
KIRP.Tumor
KIRP.Normal
LAML.Tumor
LGG.Tumor
LIHC.Tumor
LIHC.Normal
LUAD.Tumor
LUAD.Normal
LUSC.Tumor
LUSC.Normal
MESO.Tumor
OV.Tumor
PAAD.Tumor
PCPG.Tumor
PRAD.Tumor
PRAD.Normal
READ.Tumor
READ.Normal
SARC.Tumor
SKCM.Tumor
SKCM.Metastasis
STAD.Tumor
STAD.Normal
TGCT.Tumor
THCA.Tumor
THCA.Normal
THYM.Tumor
UCEC.Tumor
UCEC.Normal
UCS.Tumor
UVM.Tumor

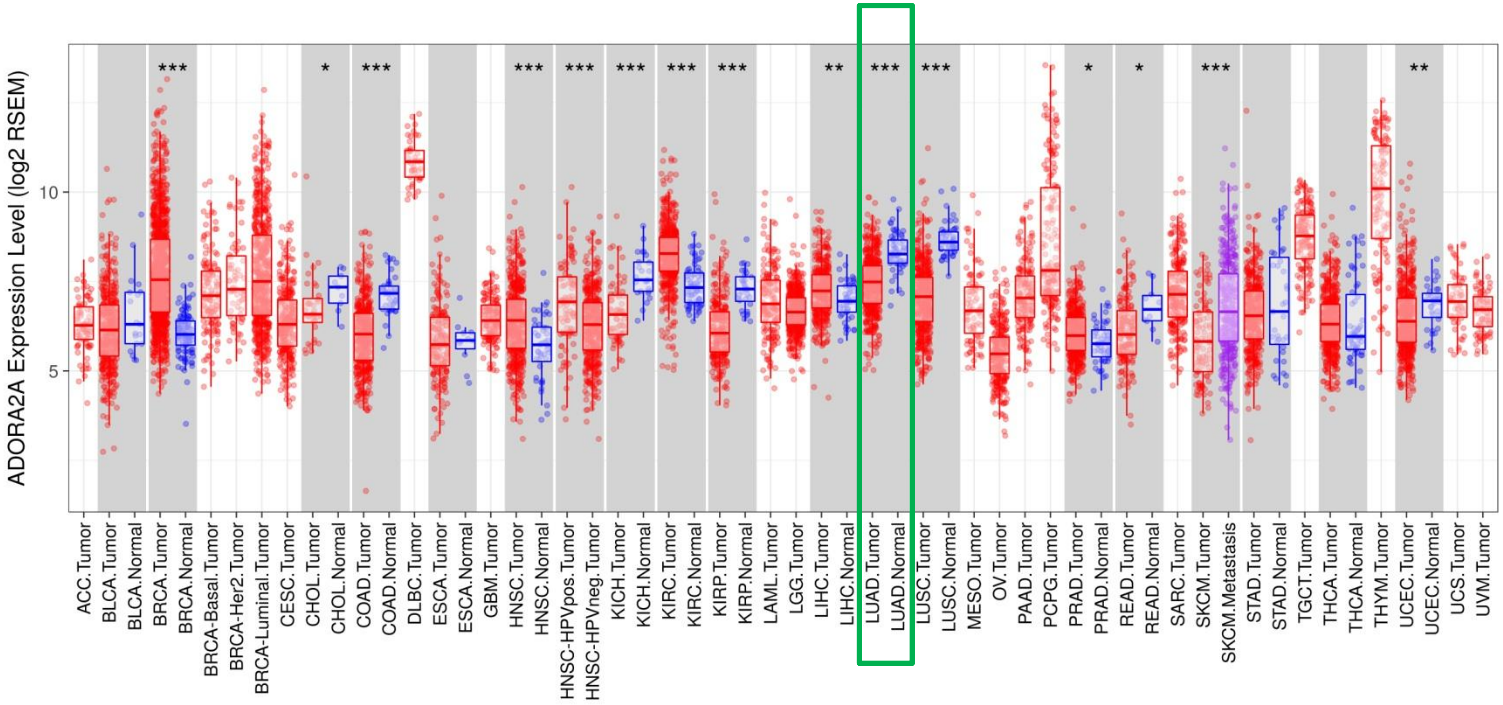

ADORA2A Expression Level (log2 RSEM)
10
5
*** * *** *** *** *** *** *** ** *** *** * * *** **
ACC.Tumor
BLCA.Tumor
BLCA.Normal
BRCA.Tumor
BRCA.Normal
BRCA-Basal.Tumor
BRCA-Her2.Tumor
BRCA-Luminal.Tumor
CESC.Tumor
CHOL.Tumor
CHOL.Normal
COAD.Tumor
COAD.Normal
DLBC.Tumor
ESCA.Tumor
ESCA.Normal
GBM.Tumor
HNSC.Tumor
HNSC.Normal
HNSC-HPVpos.Tumor
HNSC-HPVneg.Tumor
KICH.Tumor
KICH.Normal
KIRC.Tumor
KIRC.Normal
KIRP.Tumor
KIRP.Normal
LAML.Tumor
LGG.Tumor
LIHC.Tumor
LIHC.Normal
LUAD.Tumor
LUAD.Normal
LUSC.Tumor
LUSC.Normal
MESO.Tumor
OV.Tumor
PAAD.Tumor
PCPG.Tumor
PRAD.Tumor
PRAD.Normal
READ.Tumor
READ.Normal
SARC.Tumor
SKCM.Tumor
SKCM.Metastasis
STAD.Tumor
STAD.Normal
TGCT.Tumor
THCA.Tumor
THCA.Normal
THYM.Tumor
UCEC.Tumor
UCEC.Normal
UCS.Tumor
UVM.Tumor

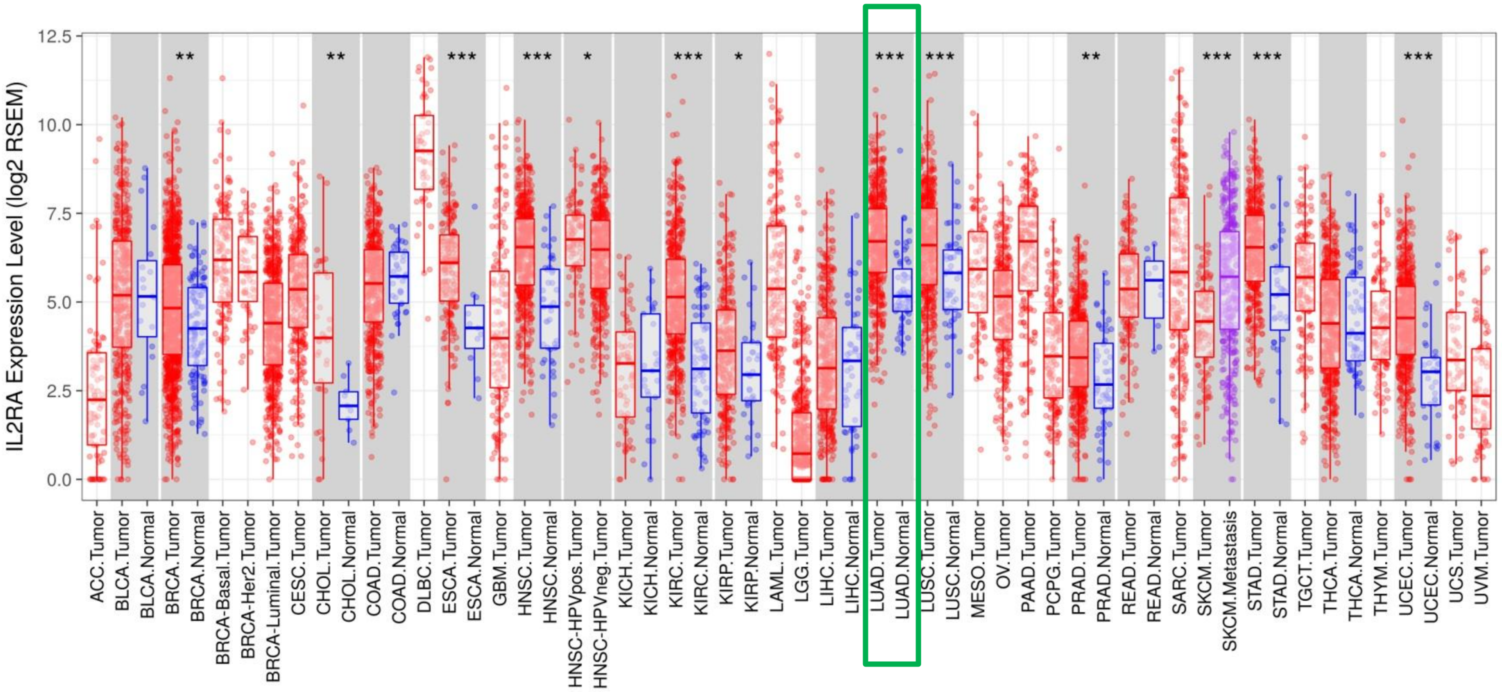
IL2RA Expression Level (log2 RSEM)
12.5
10.0
7.5
5.0
2.5
0.0
**
**
***
***
*
***
*
***
***
**
***
***
***
ACC.Tumor
BLCA.Tumor
BLCA.Normal
BRCA.Tumor
BRCA.Normal
BRCA-Basal.Tumor
BRCA-Her2.Tumor
BRCA-Luminal.Tumor
CESC.Tumor
CHOL.Tumor
CHOL.Normal
COAD.Tumor
COAD.Normal
DLBC.Tumor
ESCA.Tumor
ESCA.Normal
GBM.Tumor
HNSC.Tumor
HNSC.Normal
HNSC-HPVpos.Tumor
HNSC-HPVneg.Tumor
KICH.Tumor
KICH.Normal
KIRC.Tumor
KIRC.Normal
KIRP.Tumor
KIRP.Normal
LAML.Tumor
LGG.Tumor
LIHC.Tumor
LIHC.Normal
LUAD.Tumor
LUAD.Normal
LUSC.Tumor
LUSC.Normal
MESO.Tumor
OV.Tumor
PAAD.Tumor
PCPG.Tumor
PRAD.Tumor
PRAD.Normal
READ.Tumor
READ.Normal
SARC.Tumor
SKCM.Tumor
SKCM.Metastasis
STAD.Tumor
STAD.Normal
TGCT.Tumor
THCA.Tumor
THCA.Normal
THYM.Tumor
UCEC.Tumor
UCEC.Normal
UCS.Tumor
UVM.Tumor

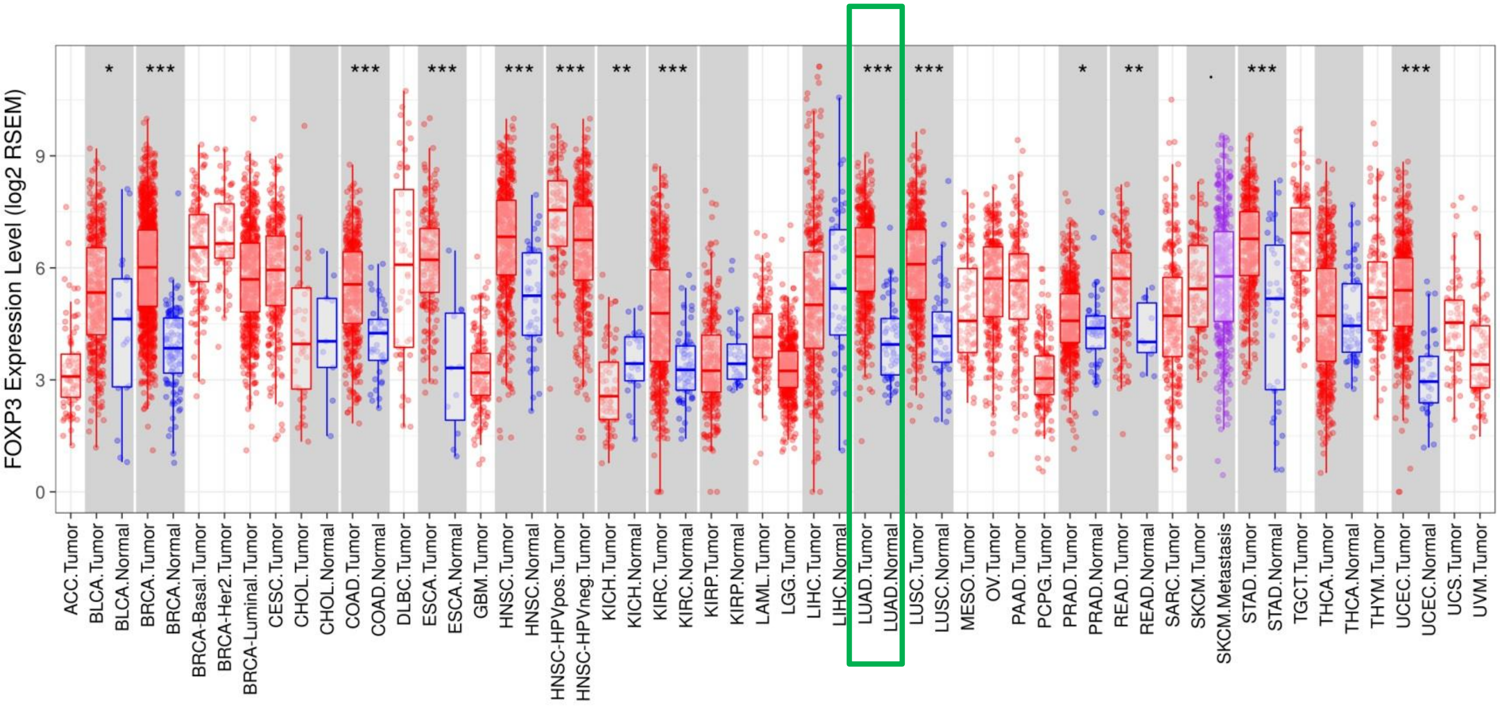
FOXP3 Expression Level (log2 RSEM)
0
3
6
9
*
***
***
***
***
***
***
**
***
***
***
*
**
.
***
***
ACC.Tumor
BLCA.Tumor
BLCA.Normal
BRCA.Tumor
BRCA.Normal
BRCA-Basal.Tumor
BRCA-Her2.Tumor
BRCA-Luminal.Tumor
CESC.Tumor
CHOL.Tumor
CHOL.Normal
COAD.Tumor
COAD.Normal
DLBC.Tumor
ESCA.Tumor
ESCA.Normal
GBM.Tumor
HNSC.Tumor
HNSC.Normal
HNSC-HPVpos.Tumor
HNSC-HPVneg.Tumor
KICH.Tumor
KICH.Normal
KIRC.Tumor
KIRC.Normal
KIRP.Tumor
KIRP.Normal
LAML.Tumor
LGG.Tumor
LIHC.Tumor
LIHC.Normal
LUAD.Tumor
LUAD.Normal
LUSC.Tumor
LUSC.Normal
MESO.Tumor
OV.Tumor
PAAD.Tumor
PCPG.Tumor
PRAD.Tumor
PRAD.Normal
READ.Tumor
READ.Normal
SARC.Tumor
SKCM.Tumor
SKCM.Metastasis
STAD.Tumor
STAD.Normal
TGCT.Tumor
THCA.Tumor
THCA.Normal
THYM.Tumor
UCEC.Tumor
UCEC.Normal
UCS.Tumor
UVM.Tumor

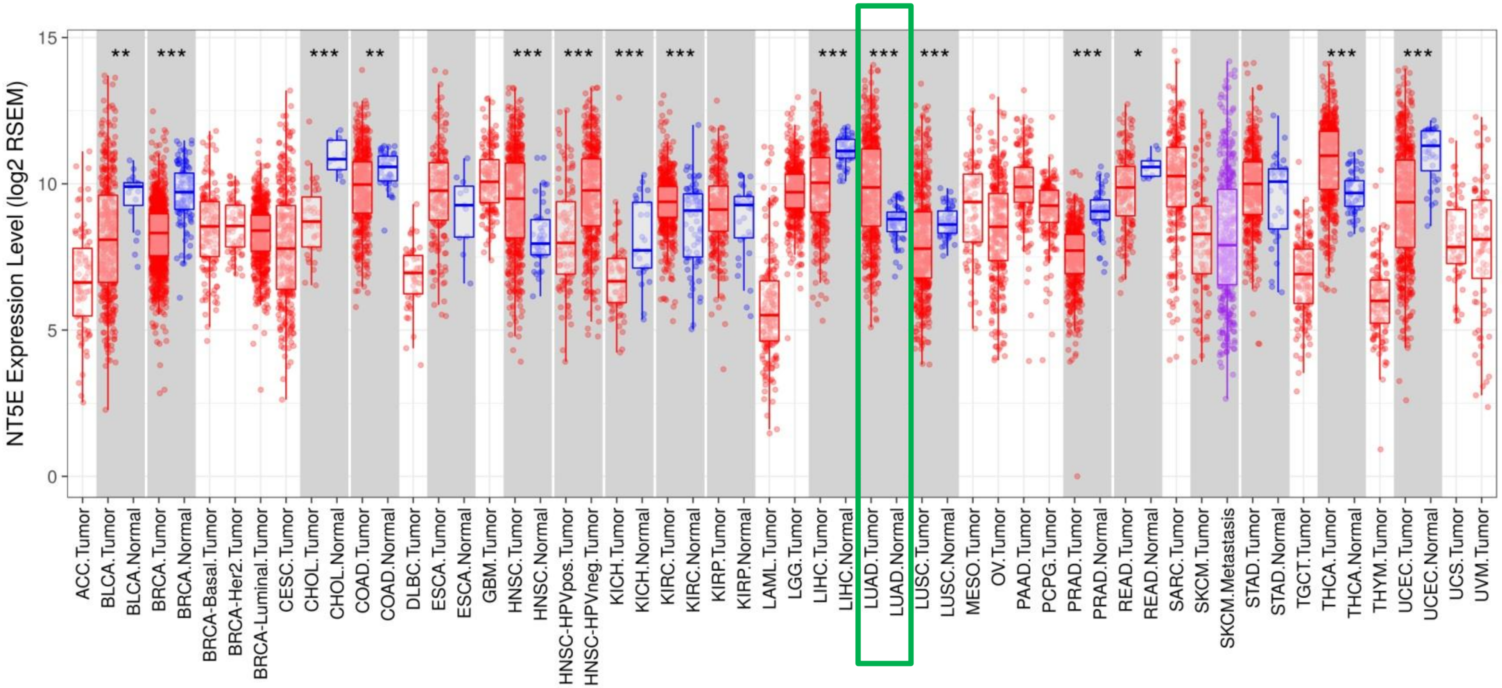
NT5E Expression Level (log2 RSEM)
15
10
5
0
ACC.Tumor
BLCA.Tumor
BLCA.Normal
BRCA.Tumor
BRCA.Normal
BRCA-Basal.Tumor
BRCA-Her2.Tumor
BRCA-Luminal.Tumor
CESC.Tumor
CHOL.Tumor
CHOL.Normal
COAD.Tumor
COAD.Normal
DLBC.Tumor
ESCA.Tumor
ESCA.Normal
GBM.Tumor
HNSC.Tumor
HNSC.Normal
HNSC-HPVpos.Tumor
HNSC-HPVneg.Tumor
KICH.Tumor
KICH.Normal
KIRC.Tumor
KIRC.Normal
KIRP.Tumor
KIRP.Normal
LAML.Tumor
LGG.Tumor
LIHC.Tumor
LIHC.Normal
LUAD.Tumor
LUAD.Normal
LUSC.Tumor
LUSC.Normal
MESO.Tumor
OV.Tumor
PAAD.Tumor
PCPG.Tumor
PRAD.Tumor
PRAD.Normal
READ.Tumor
READ.Normal
SARC.Tumor
SKCM.Tumor
SKCM.Metastasis
STAD.Tumor
STAD.Normal
TGCT.Tumor
THCA.Tumor
THCA.Normal
THYM.Tumor
UCEC.Tumor
UCEC.Normal
UCS.Tumor
UVM.Tumor

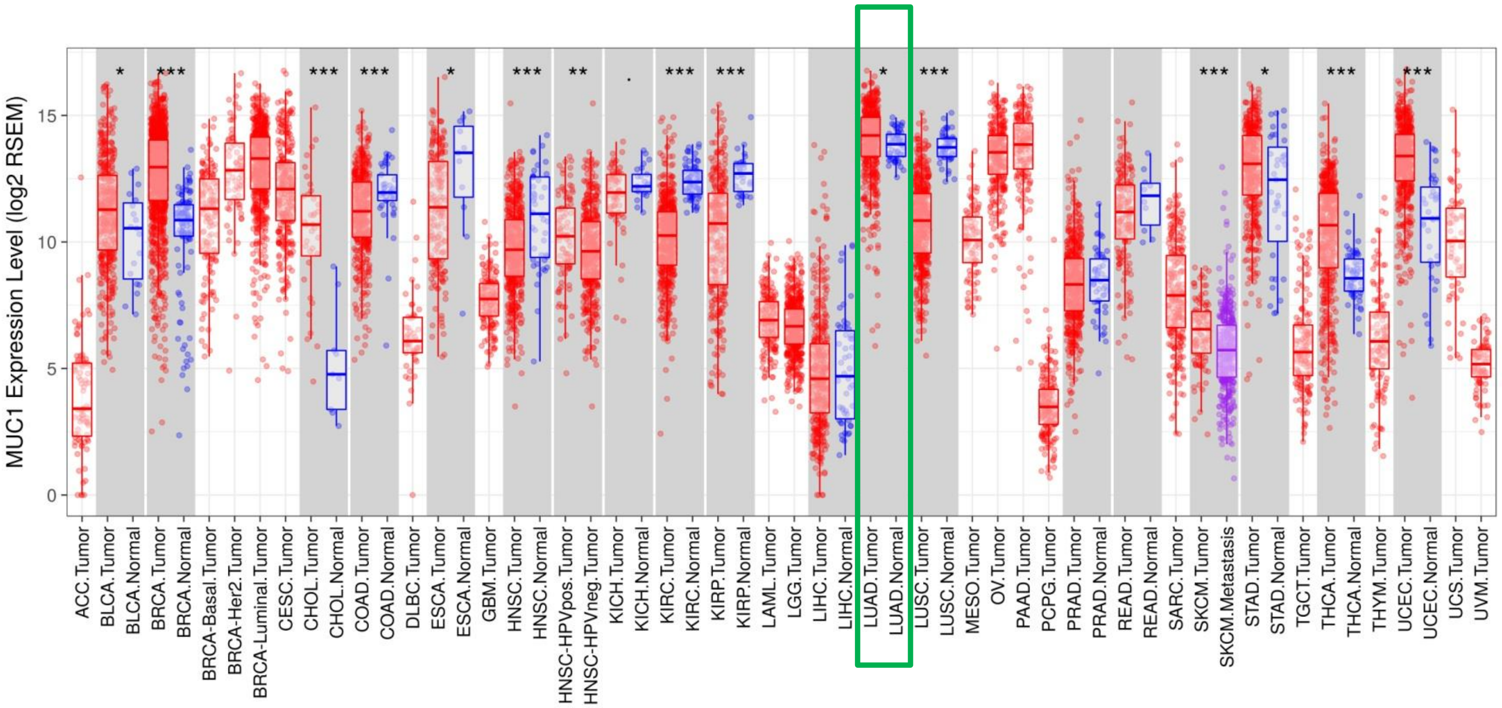
MUC1 Expression Level (log2 RSEM)
0
5
10
15
ACC.Tumor
BLCA.Tumor
BLCA.Normal
BRCA.Tumor
BRCA.Normal
BRCA-Basal.Tumor
BRCA-Her2.Tumor
BRCA-Luminal.Tumor
CESC.Tumor
CHOL.Tumor
CHOL.Normal
COAD.Tumor
COAD.Normal
DLBC.Tumor
ESCA.Tumor
ESCA.Normal
GBM.Tumor
HNSC.Tumor
HNSC.Normal
HNSC-HPVpos.Tumor
HNSC-HPVneg.Tumor
KICH.Tumor
KICH.Normal
KIRC.Tumor
KIRC.Normal
KIRP.Tumor
KIRP.Normal
LAML.Tumor
LGG.Tumor
LIHC.Tumor
LIHC.Normal
LUAD.Tumor
LUAD.Normal
LUSC.Tumor
LUSC.Normal
MESO.Tumor
OV.Tumor
PAAD.Tumor
PCPG.Tumor
PRAD.Tumor
PRAD.Normal
READ.Tumor
READ.Normal
SARC.Tumor
SKCM.Tumor
SKCM.Metastasis
STAD.Tumor
STAD.Normal
TGCT.Tumor
THCA.Tumor
THCA.Normal
THYM.Tumor
UCEC.Tumor
UCEC.Normal
UCS.Tumor
UVM.Tumor

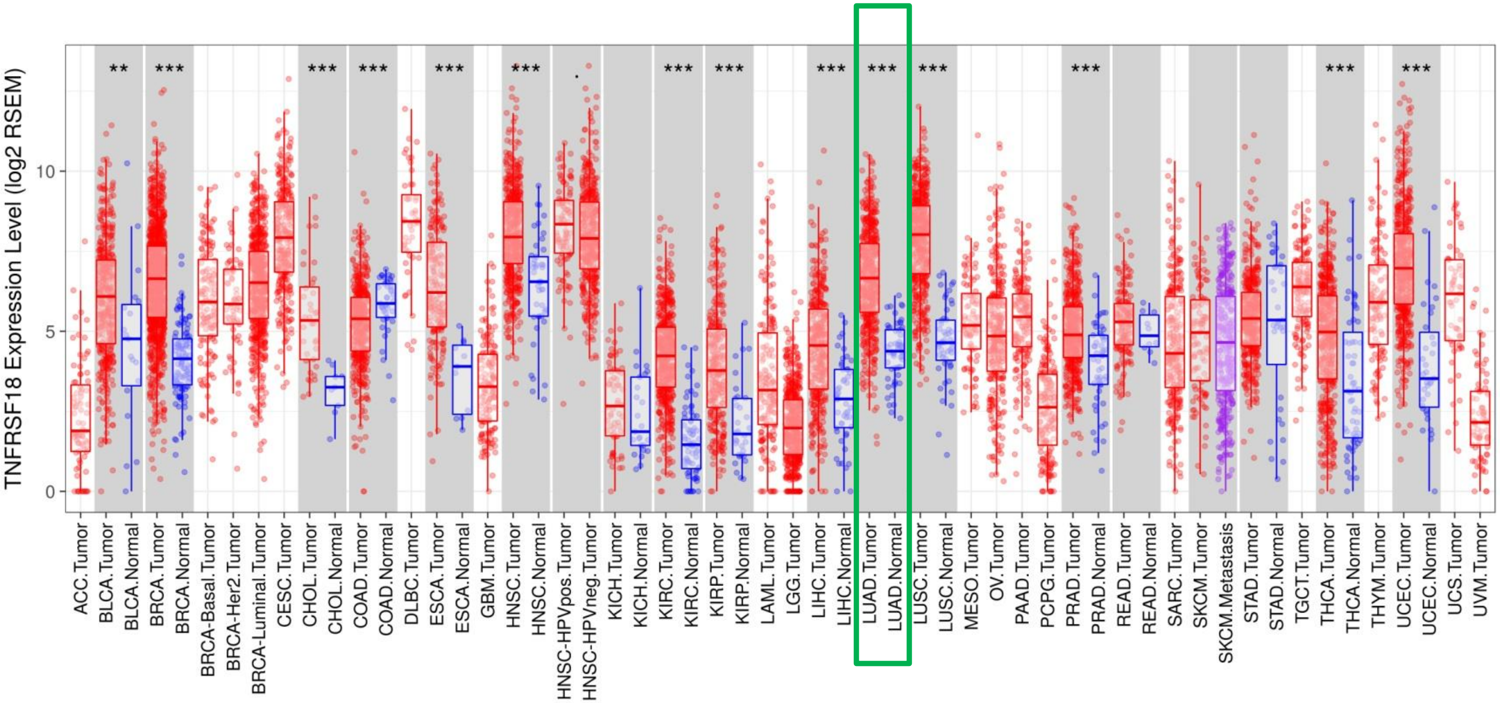

TNFRSF18 Expression Level (log2 RSEM)
0
5
10
**
***
***
***
***
***
***
***
***
***
***
***
***
***
ACC.Tumor
BLCA.Tumor
BLCA.Normal
BRCA.Tumor
BRCA.Normal
BRCA-Basal.Tumor
BRCA-Her2.Tumor
BRCA-Luminal.Tumor
CESC.Tumor
CHOL.Tumor
CHOL.Normal
COAD.Tumor
COAD.Normal
DLBC.Tumor
ESCA.Tumor
ESCA.Normal
GBM.Tumor
HNSC.Tumor
HNSC.Normal
HNSC-HPVpos.Tumor
HNSC-HPVneg.Tumor
KICH.Tumor
KICH.Normal
KIRC.Tumor
KIRC.Normal
KIRP.Tumor
KIRP.Normal
LAML.Tumor
LGG.Tumor
LIHC.Tumor
LIHC.Normal
LUAD.Tumor
LUAD.Normal
LUSC.Tumor
LUSC.Normal
MESO.Tumor
OV.Tumor
PAAD.Tumor
PCPG.Tumor
PRAD.Tumor
PRAD.Normal
READ.Tumor
READ.Normal
SARC.Tumor
SKCM.Tumor
SKCM.Metastasis
STAD.Tumor
STAD.Normal
TGCT.Tumor
THCA.Tumor
THCA.Normal
THYM.Tumor
UCEC.Tumor
UCEC.Normal
UCS.Tumor
UVM.Tumor

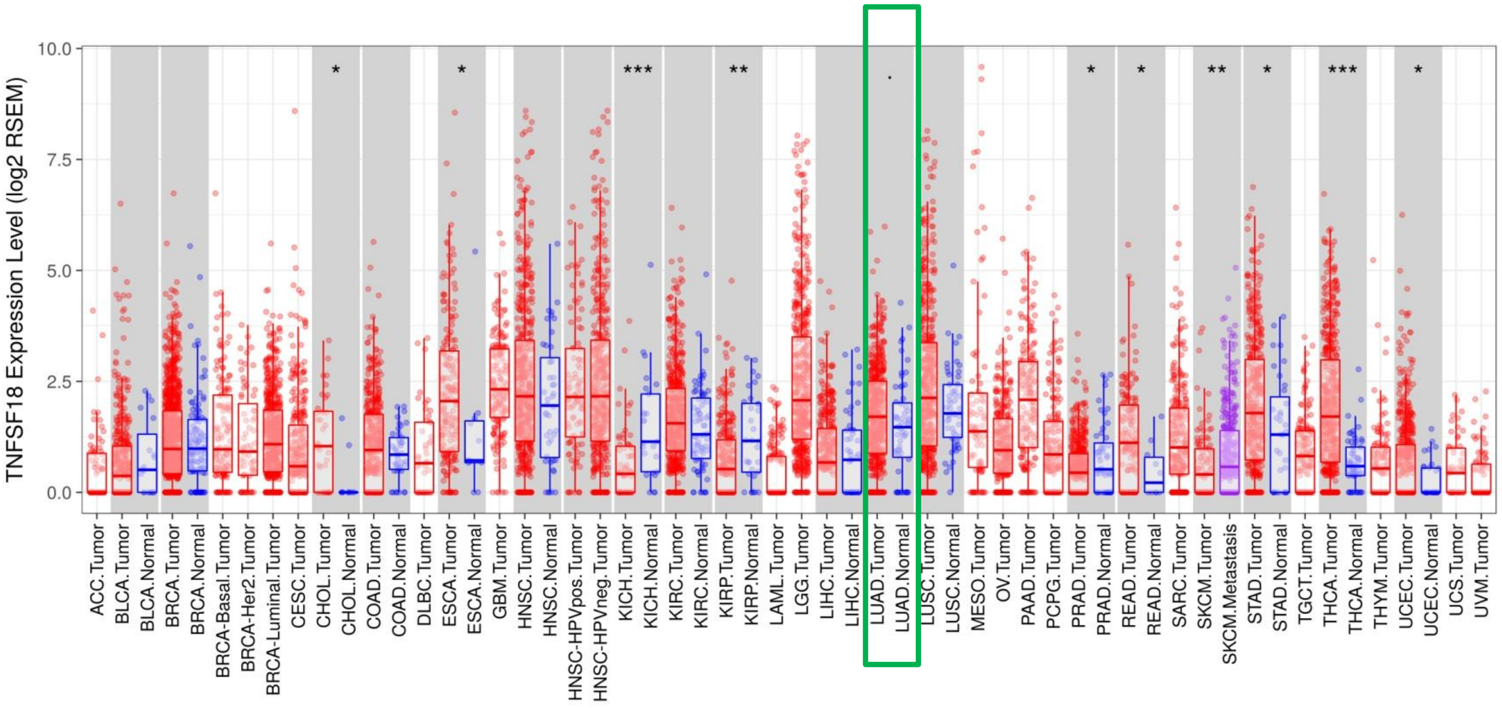
TNFSF18 Expression Level (log2 RSEM)
10.0
7.5
5.0
2.5
0.0
*
*
***
**
.
*
*
**
*
***
*
ACC.Tumor
BLCA.Tumor
BLCA.Normal
BRCA.Tumor
BRCA.Normal
BRCA-Basal.Tumor
BRCA-Her2.Tumor
BRCA-Luminal.Tumor
CESC.Tumor
CHOL.Tumor
CHOL.Normal
COAD.Tumor
COAD.Normal
DLBC.Tumor
ESCA.Tumor
ESCA.Normal
GBM.Tumor
HNSC.Tumor
HNSC.Normal
HNSC-HPVpos.Tumor
HNSC-HPVneg.Tumor
KICH.Tumor
KICH.Normal
KIRC.Tumor
KIRC.Normal
KIRP.Tumor
KIRP.Normal
LAML.Tumor
LGG.Tumor
LIHC.Tumor
LIHC.Normal
LUAD.Tumor
LUAD.Normal
LUSC.Tumor
LUSC.Normal
MESO.Tumor
OV.Tumor
PAAD.Tumor
PCPG.Tumor
PRAD.Tumor
PRAD.Normal
READ.Tumor
READ.Normal
SARC.Tumor
SKCM.Tumor
SKCM.Metastasis
STAD.Tumor
STAD.Normal
TGCT.Tumor
THCA.Tumor
THCA.Normal
THYM.Tumor
UCEC.Tumor
UCEC.Normal
UCS.Tumor
UVM.Tumor

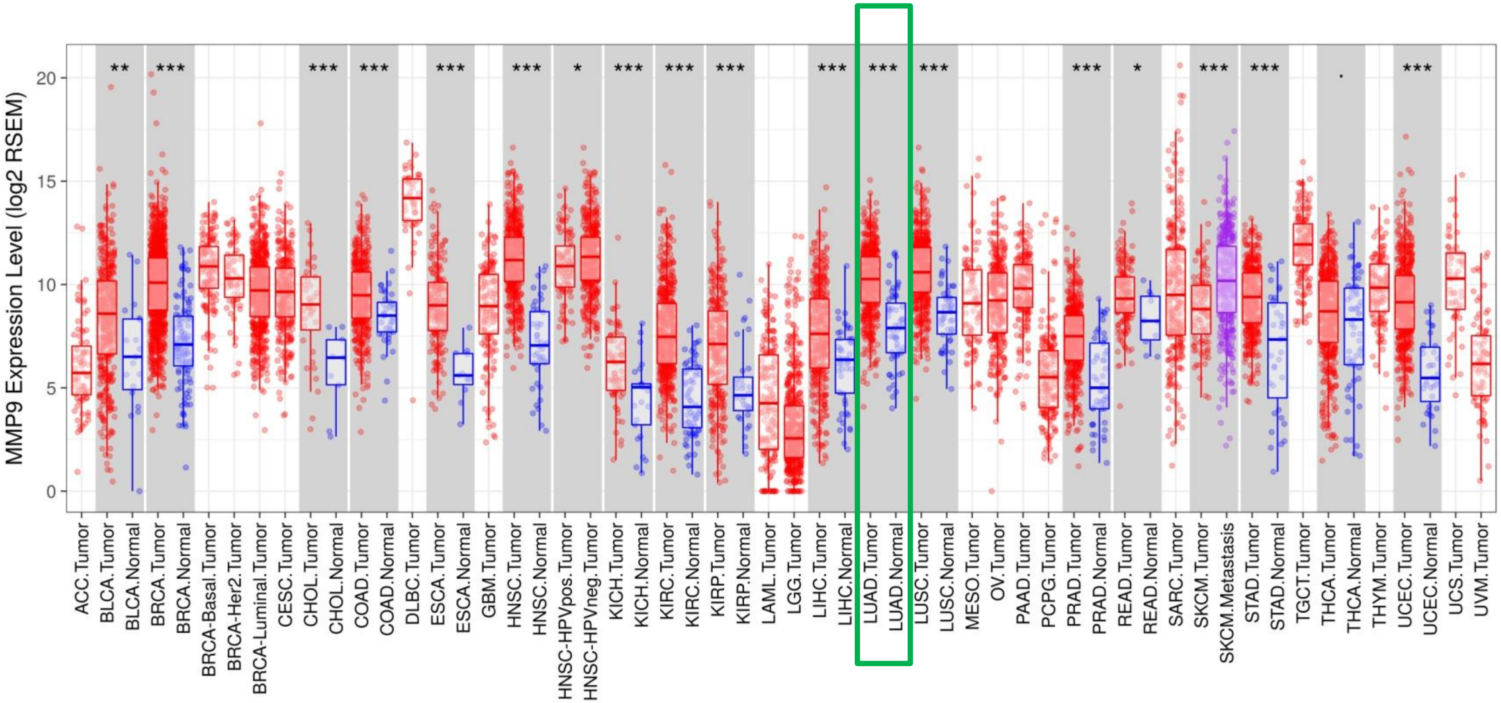
MMP9 Expression Level (log2 RSEM)
20
15
10
5
0
**
***
***
***
***
***
*
***
***
***
***
***
***
***
*
***
***
.
***
ACC.Tumor
BLCA.Tumor
BLCA.Normal
BRCA.Tumor
BRCA.Normal
BRCA-Basal.Tumor
BRCA-Her2.Tumor
BRCA-Luminal.Tumor
CESC.Tumor
CHOL.Tumor
CHOL.Normal
COAD.Tumor
COAD.Normal
DLBC.Tumor
ESCA.Tumor
ESCA.Normal
GBM.Tumor
HNSC.Tumor
HNSC.Normal
HNSC-HPVpos.Tumor
HNSC-HPVneg.Tumor
KICH.Tumor
KICH.Normal
KIRC.Tumor
KIRC.Normal
KIRP.Tumor
KIRP.Normal
LAML.Tumor
LGG.Tumor
LIHC.Tumor
LIHC.Normal
LUAD.Tumor
LUAD.Normal
LUSC.Tumor
LUSC.Normal
MESO.Tumor
OV.Tumor
PAAD.Tumor
PCPG.Tumor
PRAD.Tumor
PRAD.Normal
READ.Tumor
READ.Normal
SARC.Tumor
SKCM.Tumor
SKCM.Metastasis
STAD.Tumor
STAD.Normal
TGCT.Tumor
THCA.Tumor
THCA.Normal
THYM.Tumor
UCEC.Tumor
UCEC.Normal
UCS.Tumor
UVM.Tumor

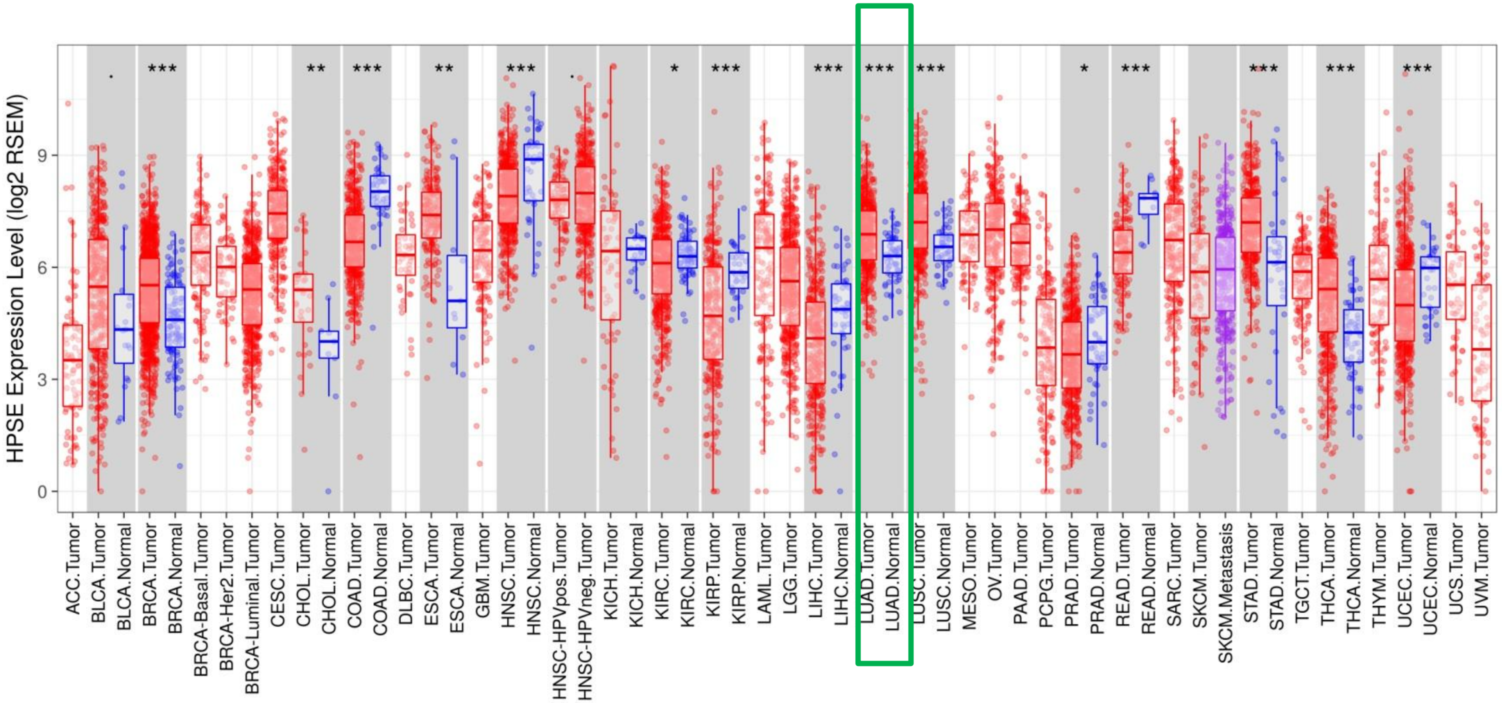
HPSE Expression Level (log2 RSEM)
0
3
6
9
ACC.Tumor
BLCA.Tumor
BLCA.Normal
BRCA.Tumor
BRCA.Normal
BRCA-Basal.Tumor
BRCA-Her2.Tumor
BRCA-Luminal.Tumor
CESC.Tumor
CHOL.Tumor
CHOL.Normal
COAD.Tumor
COAD.Normal
DLBC.Tumor
ESCA.Tumor
ESCA.Normal
GBM.Tumor
HNSC.Tumor
HNSC.Normal
HNSC-HPVpos.Tumor
HNSC-HPVneg.Tumor
KICH.Tumor
KICH.Normal
KIRC.Tumor
KIRC.Normal
KIRP.Tumor
KIRP.Normal
LAML.Tumor
LGG.Tumor
LIHC.Tumor
LIHC.Normal
LUAD.Tumor
LUAD.Normal
LUSC.Tumor
LUSC.Normal
MESO.Tumor
OV.Tumor
PAAD.Tumor
PCPG.Tumor
PRAD.Tumor
PRAD.Normal
READ.Tumor
READ.Normal
SARC.Tumor
SKCM.Tumor
SKCM.Metastasis
STAD.Tumor
STAD.Normal
TGCT.Tumor
THCA.Tumor
THCA.Normal
THYM.Tumor
UCEC.Tumor
UCEC.Normal
UCS.Tumor
UVM.Tumor

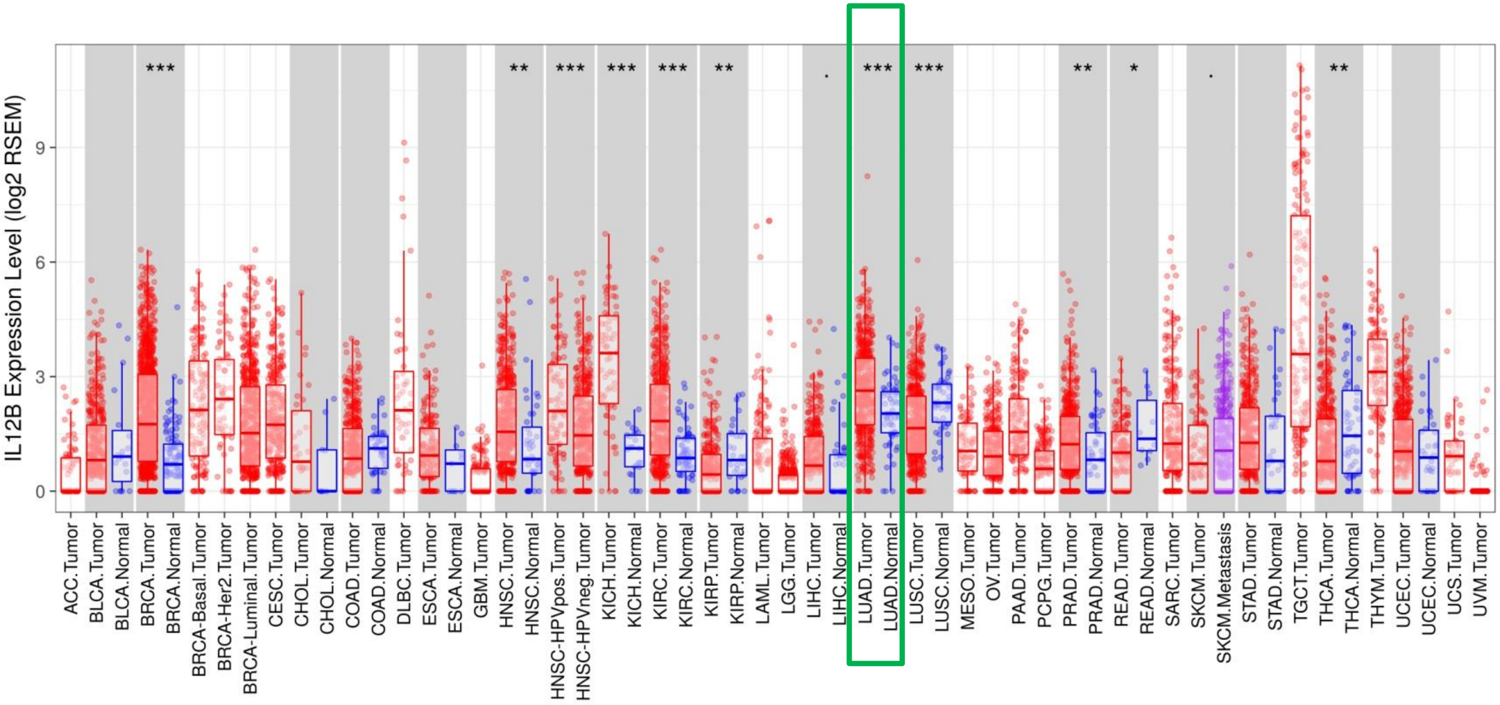
IL12B Expression Level (log2 RSEM)
0
3
6
9
***
**
***
***
***
**
.
***
***
**
*
.
**
ACC.Tumor
BLCA.Tumor
BLCA.Normal
BRCA.Tumor
BRCA.Normal
BRCA-Basal.Tumor
BRCA-Her2.Tumor
BRCA-Luminal.Tumor
CESC.Tumor
CHOL.Tumor
CHOL.Normal
COAD.Tumor
COAD.Normal
DLBC.Tumor
ESCA.Tumor
ESCA.Normal
GBM.Tumor
HNSC.Tumor
HNSC.Normal
HNSC-HPVpos.Tumor
HNSC-HPVneg.Tumor
KICH.Tumor
KICH.Normal
KIRC.Tumor
KIRC.Normal
KIRP.Tumor
KIRP.Normal
LAML.Tumor
LGG.Tumor
LIHC.Tumor
LIHC.Normal
LUAD.Tumor
LUAD.Normal
LUSC.Tumor
LUSC.Normal
MESO.Tumor
OV.Tumor
PAAD.Tumor
PCPG.Tumor
PRAD.Tumor
PRAD.Normal
READ.Tumor
READ.Normal
SARC.Tumor
SKCM.Tumor
SKCM.Metastasis
STAD.Tumor
STAD.Normal
TGCT.Tumor
THCA.Tumor
THCA.Normal
THYM.Tumor
UCEC.Tumor
UCEC.Normal
UCS.Tumor
UVM.Tumor

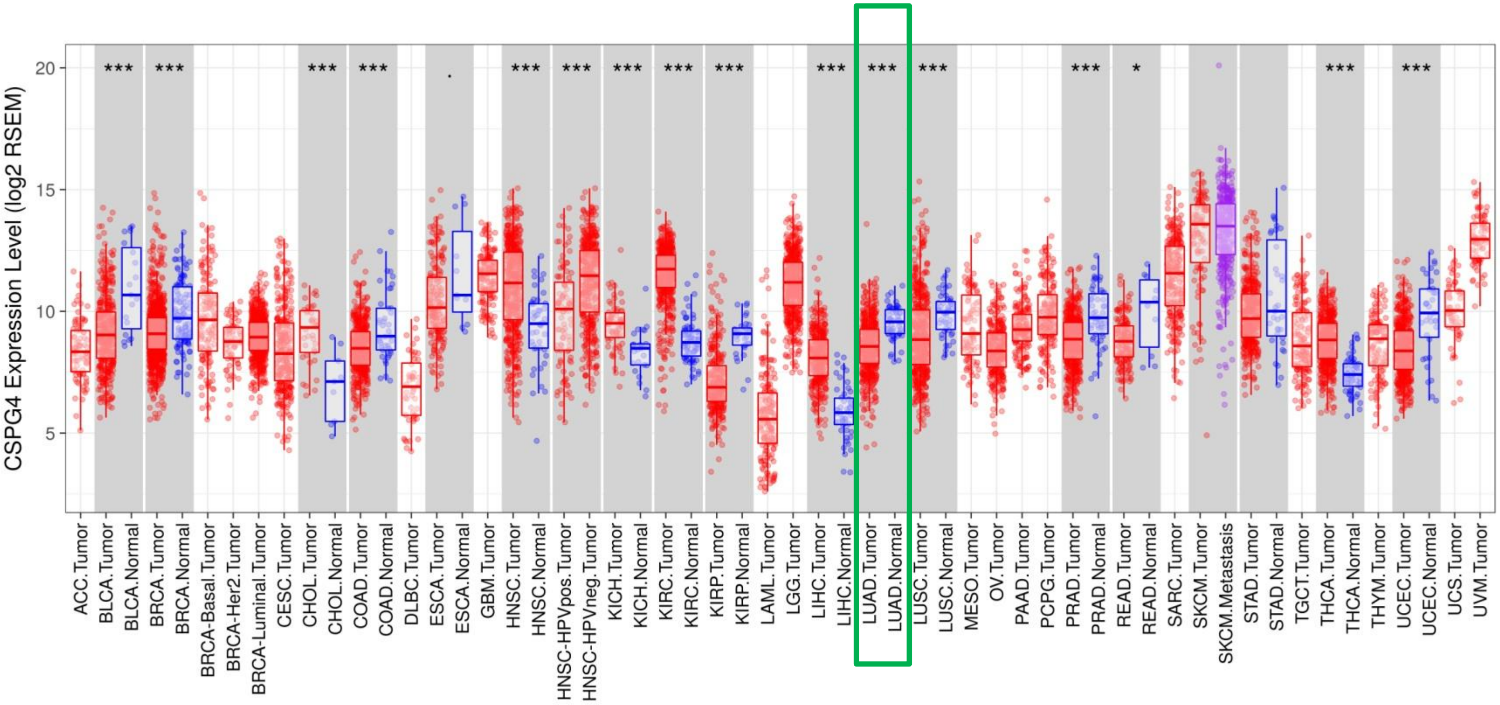
CSPG4 Expression Level (log2 RSEM)
20
15
10
5
***
***
***
***
.
***
***
***
***
***
***
***
***
***
*
***
***
ACC.Tumor
BLCA.Tumor
BLCA.Normal
BRCA.Tumor
BRCA.Normal
BRCA-Basal.Tumor
BRCA-Her2.Tumor
BRCA-Luminal.Tumor
CESC.Tumor
CHOL.Tumor
CHOL.Normal
COAD.Tumor
COAD.Normal
DLBC.Tumor
ESCA.Tumor
ESCA.Normal
GBM.Tumor
HNSC.Tumor
HNSC.Normal
HNSC-HPVpos.Tumor
HNSC-HPVneg.Tumor
KICH.Tumor
KICH.Normal
KIRC.Tumor
KIRC.Normal
KIRP.Tumor
KIRP.Normal
LAML.Tumor
LGG.Tumor
LIHC.Tumor
LIHC.Normal
LUAD.Tumor
LUAD.Normal
LUSC.Tumor
LUSC.Normal
MESO.Tumor
OV.Tumor
PAAD.Tumor
PCPG.Tumor
PRAD.Tumor
PRAD.Normal
READ.Tumor
READ.Normal
SARC.Tumor
SKCM.Tumor
SKCM.Metastasis
STAD.Tumor
STAD.Normal
TGCT.Tumor
THCA.Tumor
THCA.Normal
THYM.Tumor
UCEC.Tumor
UCEC.Normal
UCS.Tumor
UVM.Tumor

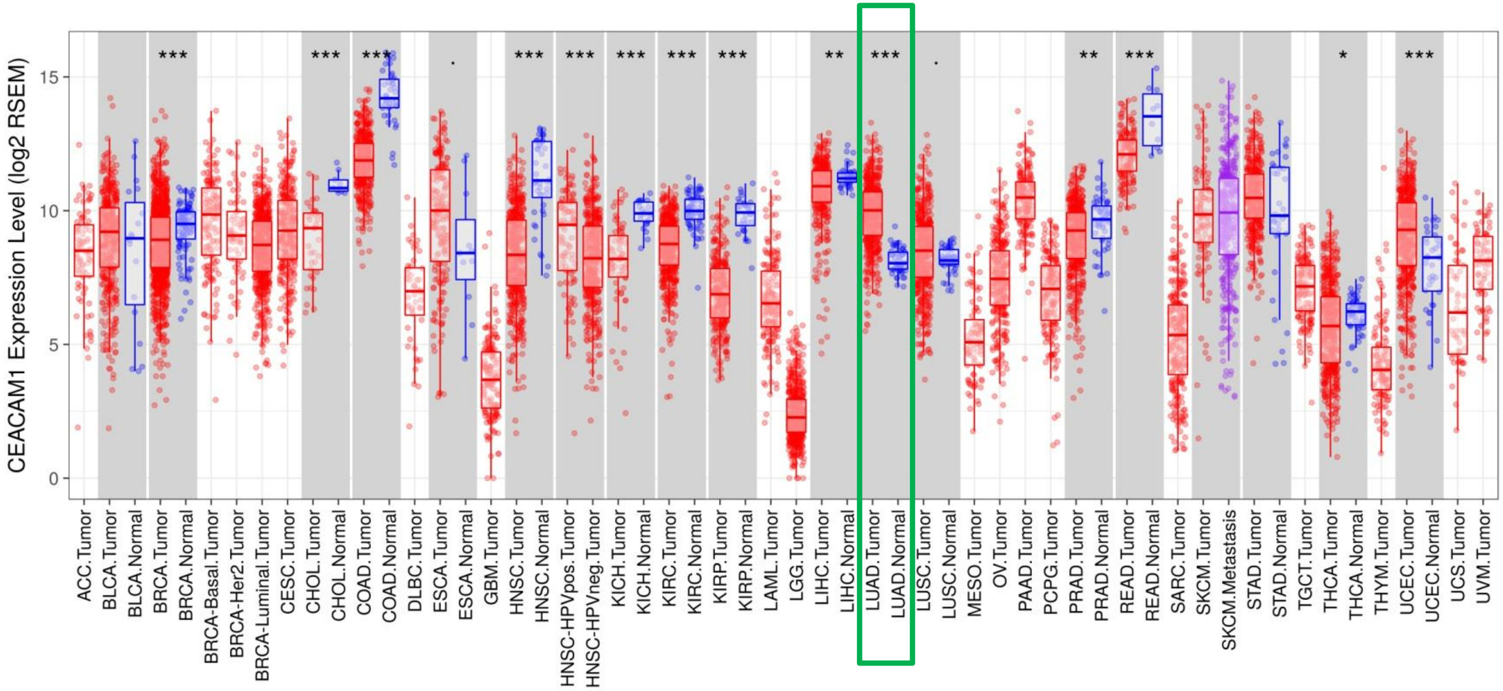
CEACAM1 Expression Level (log2 RSEM)
0
5
10
15
*** *** *** . *** *** *** *** *** ** *** . ** *** * ***
ACC.Tumor
BLCA.Tumor
BLCA.Normal
BRCA.Tumor
BRCA.Normal
BRCA-Basal.Tumor
BRCA-Her2.Tumor
BRCA-Luminal.Tumor
CESC.Tumor
CHOL.Tumor
CHOL.Normal
COAD.Tumor
COAD.Normal
DLBC.Tumor
ESCA.Tumor
ESCA.Normal
GBM.Tumor
HNSC.Tumor
HNSC.Normal
HNSC-HPVpos.Tumor
HNSC-HPVneg.Tumor
KICH.Tumor
KICH.Normal
KIRC.Tumor
KIRC.Normal
KIRP.Tumor
KIRP.Normal
LAML.Tumor
LGG.Tumor
LIHC.Tumor
LIHC.Normal
LUAD.Tumor
LUAD.Normal
LUSC.Tumor
LUSC.Normal
MESO.Tumor
OV.Tumor
PAAD.Tumor
PCPG.Tumor
PRAD.Tumor
PRAD.Normal
READ.Tumor
READ.Normal
SARC.Tumor
SKCM.Tumor
SKCM.Metastasis
STAD.Tumor
STAD.Normal
TGCT.Tumor
THCA.Tumor
THCA.Normal
THYM.Tumor
UCEC.Tumor
UCEC.Normal
UCS.Tumor
UVM.Tumor

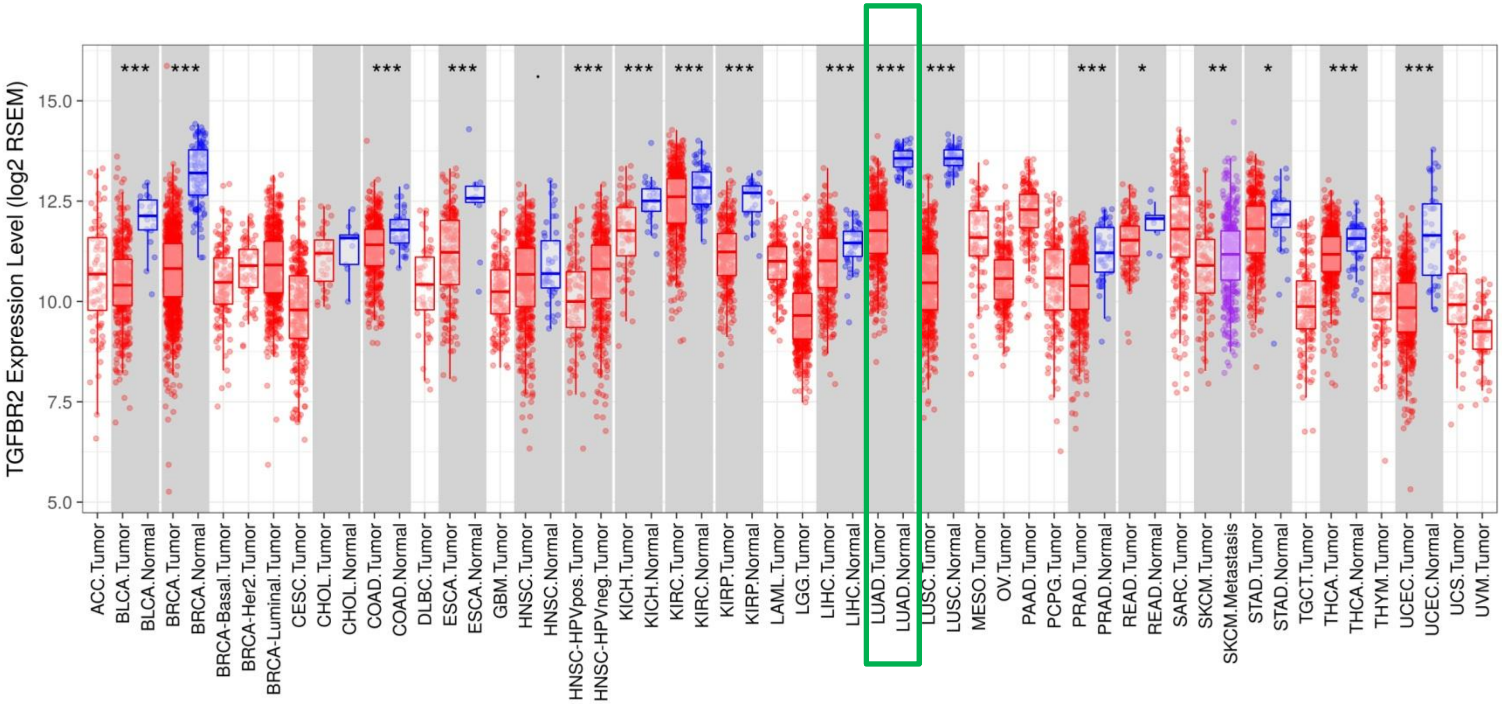

TGFBR2 Expression Level (log2 RSEM)
15.0
12.5
10.0
7.5
5.0
***
***
***
***
.
***
***
***
***
***
***
***
***
*
**
*
***
***
ACC.Tumor
BLCA.Tumor
BLCA.Normal
BRCA.Tumor
BRCA.Normal
BRCA-Basal.Tumor
BRCA-Her2.Tumor
BRCA-Luminal.Tumor
CESC.Tumor
CHOL.Tumor
CHOL.Normal
COAD.Tumor
COAD.Normal
DLBC.Tumor
ESCA.Tumor
ESCA.Normal
GBM.Tumor
HNSC.Tumor
HNSC.Normal
HNSC-HPVpos.Tumor
HNSC-HPVneg.Tumor
KICH.Tumor
KICH.Normal
KIRC.Tumor
KIRC.Normal
KIRP.Tumor
KIRP.Normal
LAML.Tumor
LGG.Tumor
LIHC.Tumor
LIHC.Normal
LUAD.Tumor
LUAD.Normal
LUSC.Tumor
LUSC.Normal
MESO.Tumor
OV.Tumor
PAAD.Tumor
PCPG.Tumor
PRAD.Tumor
PRAD.Normal
READ.Tumor
READ.Normal
SARC.Tumor
SKCM.Tumor
SKCM.Metastasis
STAD.Tumor
STAD.Normal
TGCT.Tumor
THCA.Tumor
THCA.Normal
THYM.Tumor
UCEC.Tumor
UCEC.Normal
UCS.Tumor
UVM.Tumor

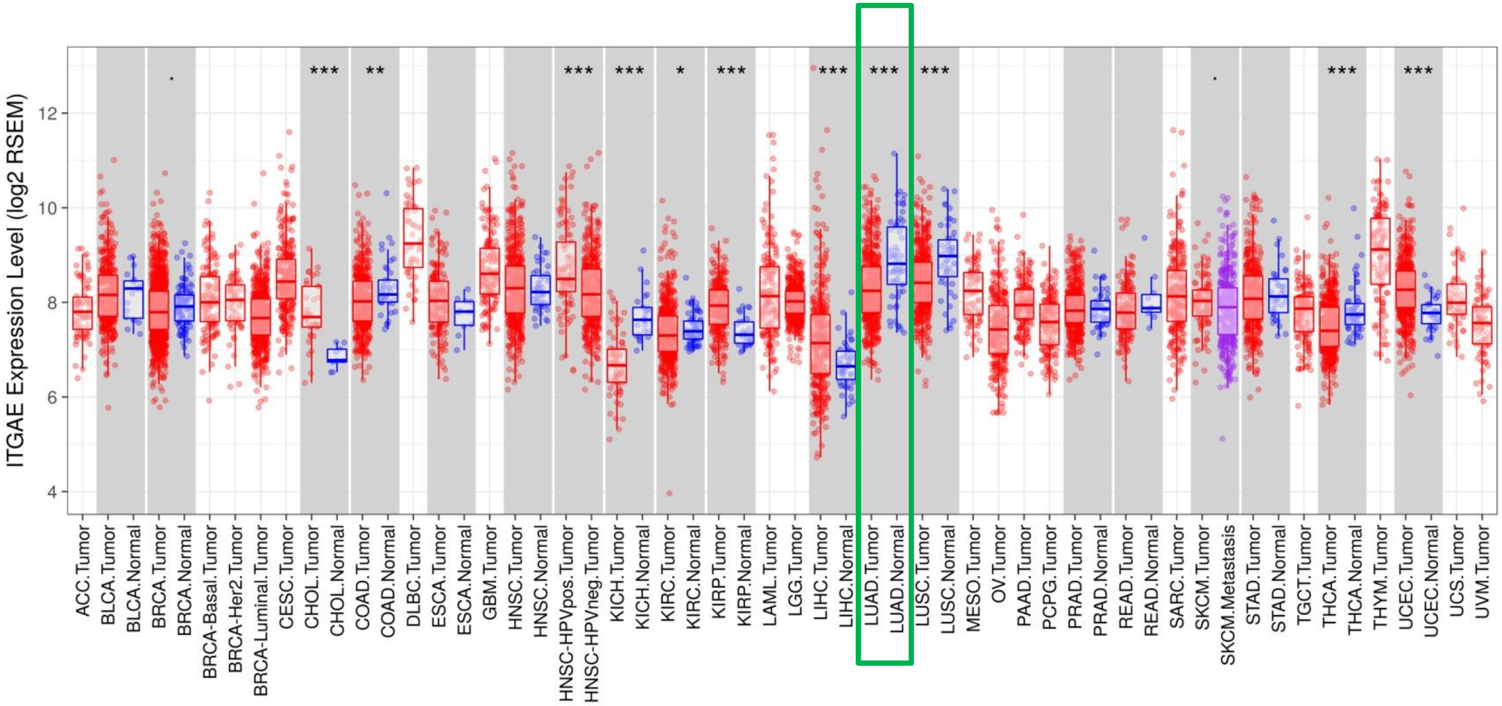

ITGAE Expression Level (log2 RSEM)
12
10
8
6
4
***
**
***
***
*
***
***
***
***
***
***
ACC.Tumor
BLCA.Tumor
BLCA.Normal
BRCA.Tumor
BRCA.Normal
BRCA-Basal.Tumor
BRCA-Her2.Tumor
BRCA-Luminal.Tumor
CESC.Tumor
CHOL.Tumor
CHOL.Normal
COAD.Tumor
COAD.Normal
DLBC.Tumor
ESCA.Tumor
ESCA.Normal
GBM.Tumor
HNSC.Tumor
HNSC.Normal
HNSC-HPVpos.Tumor
HNSC-HPVneg.Tumor
KICH.Tumor
KICH.Normal
KIRC.Tumor
KIRC.Normal
KIRP.Tumor
KIRP.Normal
LAML.Tumor
LGG.Tumor
LIHC.Tumor
LIHC.Normal
LUAD.Tumor
LUAD.Normal
LUSC.Tumor
LUSC.Normal
MESO.Tumor
OV.Tumor
PAAD.Tumor
PCPG.Tumor
PRAD.Tumor
PRAD.Normal
READ.Tumor
READ.Normal
SARC.Tumor
SKCM.Tumor
SKCM.Metastasis
STAD.Tumor
STAD.Normal
TGCT.Tumor
THCA.Tumor
THCA.Normal
THYM.Tumor
UCEC.Tumor
UCEC.Normal
UCS.Tumor
UVM.Tumor

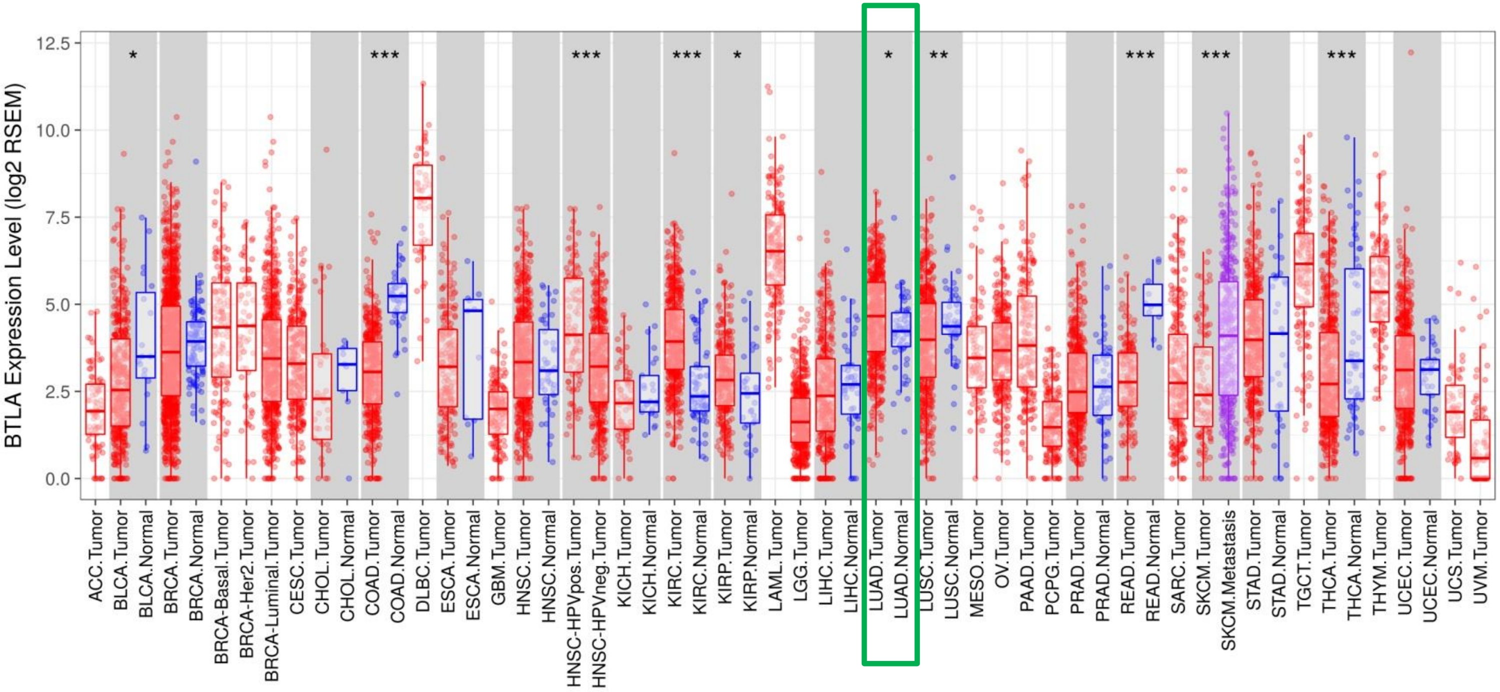
BTLA Expression Level (log2 RSEM)
12.5
10.0
7.5
5.0
2.5
0.0
*
***
***
***
*
*
**
***
***
***
ACC.Tumor
BLCA.Tumor
BLCA.Normal
BRCA.Tumor
BRCA.Normal
BRCA-Basal.Tumor
BRCA-Her2.Tumor
BRCA-Luminal.Tumor
CESC.Tumor
CHOL.Tumor
CHOL.Normal
COAD.Tumor
COAD.Normal
DLBC.Tumor
ESCA.Tumor
ESCA.Normal
GBM.Tumor
HNSC.Tumor
HNSC.Normal
HNSC-HPVpos.Tumor
HNSC-HPVneg.Tumor
KICH.Tumor
KICH.Normal
KIRC.Tumor
KIRC.Normal
KIRP.Tumor
KIRP.Normal
LAML.Tumor
LGG.Tumor
LIHC.Tumor
LIHC.Normal
LUAD.Tumor
LUAD.Normal
LUSC.Tumor
LUSC.Normal
MESO.Tumor
OV.Tumor
PAAD.Tumor
PCPG.Tumor
PRAD.Tumor
PRAD.Normal
READ.Tumor
READ.Normal
SARC.Tumor
SKCM.Tumor
SKCM.Metastasis
STAD.Tumor
STAD.Normal
TGCT.Tumor
THCA.Tumor
THCA.Normal
THYM.Tumor
UCEC.Tumor
UCEC.Normal
UCS.Tumor
UVM.Tumor

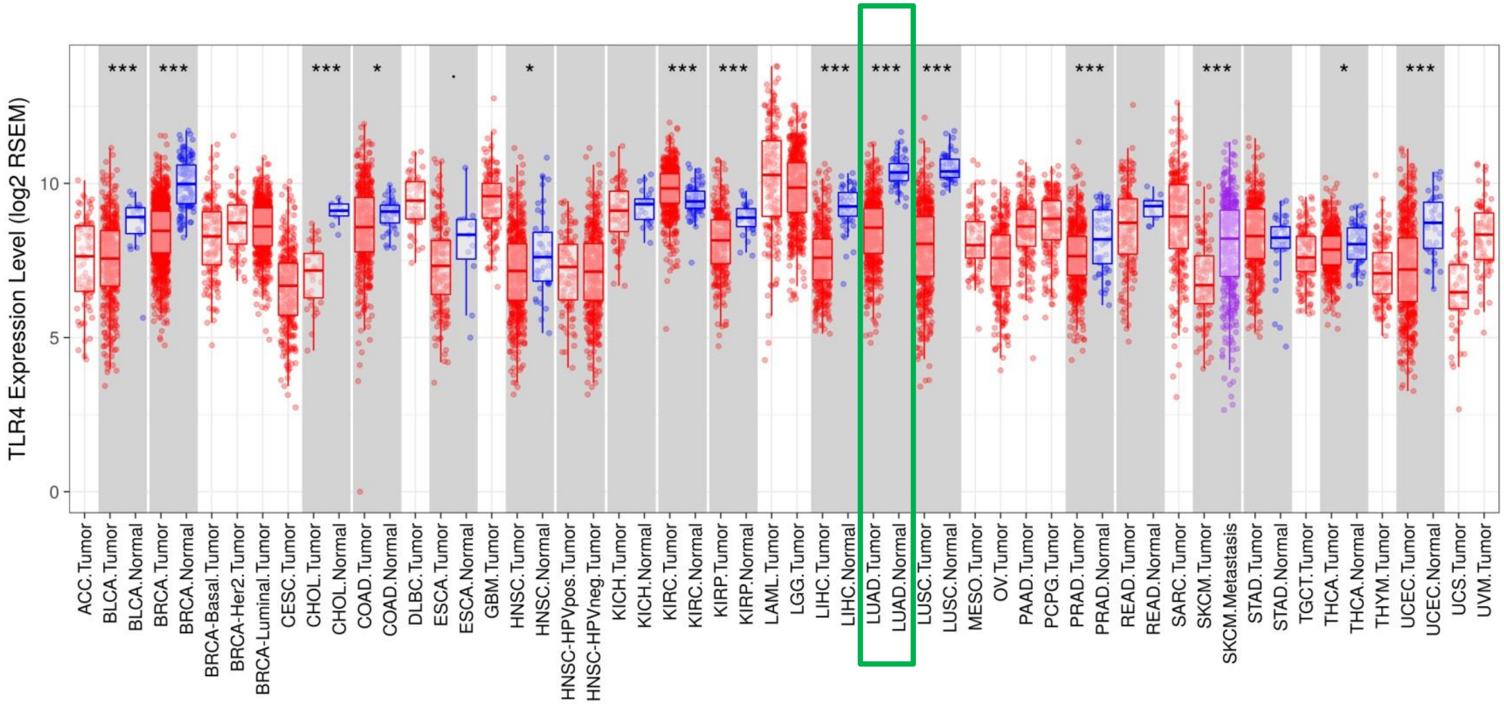
TLR4 Expression Level (log2 RSEM)
0
5
10
*** *** *** * . * *** *** *** *** *** *** *** * ***
ACC.Tumor
BLCA.Tumor
BLCA.Normal
BRCA.Tumor
BRCA.Normal
BRCA-Basal.Tumor
BRCA-Her2.Tumor
BRCA-Luminal.Tumor
CESC.Tumor
CHOL.Tumor
CHOL.Normal
COAD.Tumor
COAD.Normal
DLBC.Tumor
ESCA.Tumor
ESCA.Normal
GBM.Tumor
HNSC.Tumor
HNSC.Normal
HNSC-HPVpos.Tumor
HNSC-HPVneg.Tumor
KICH.Tumor
KICH.Normal
KIRC.Tumor
KIRC.Normal
KIRP.Tumor
KIRP.Normal
LAML.Tumor
LGG.Tumor
LIHC.Tumor
LIHC.Normal
LUAD.Tumor
LUAD.Normal
LUSC.Tumor
LUSC.Normal
MESO.Tumor
OV.Tumor
PAAD.Tumor
PCPG.Tumor
PRAD.Tumor
PRAD.Normal
READ.Tumor
READ.Normal
SARC.Tumor
SKCM.Tumor
SKCM.Metastasis
STAD.Tumor
STAD.Normal
TGCT.Tumor
THCA.Tumor
THCA.Normal
THYM.Tumor
UCEC.Tumor
UCEC.Normal
UCS.Tumor
UVM.Tumor

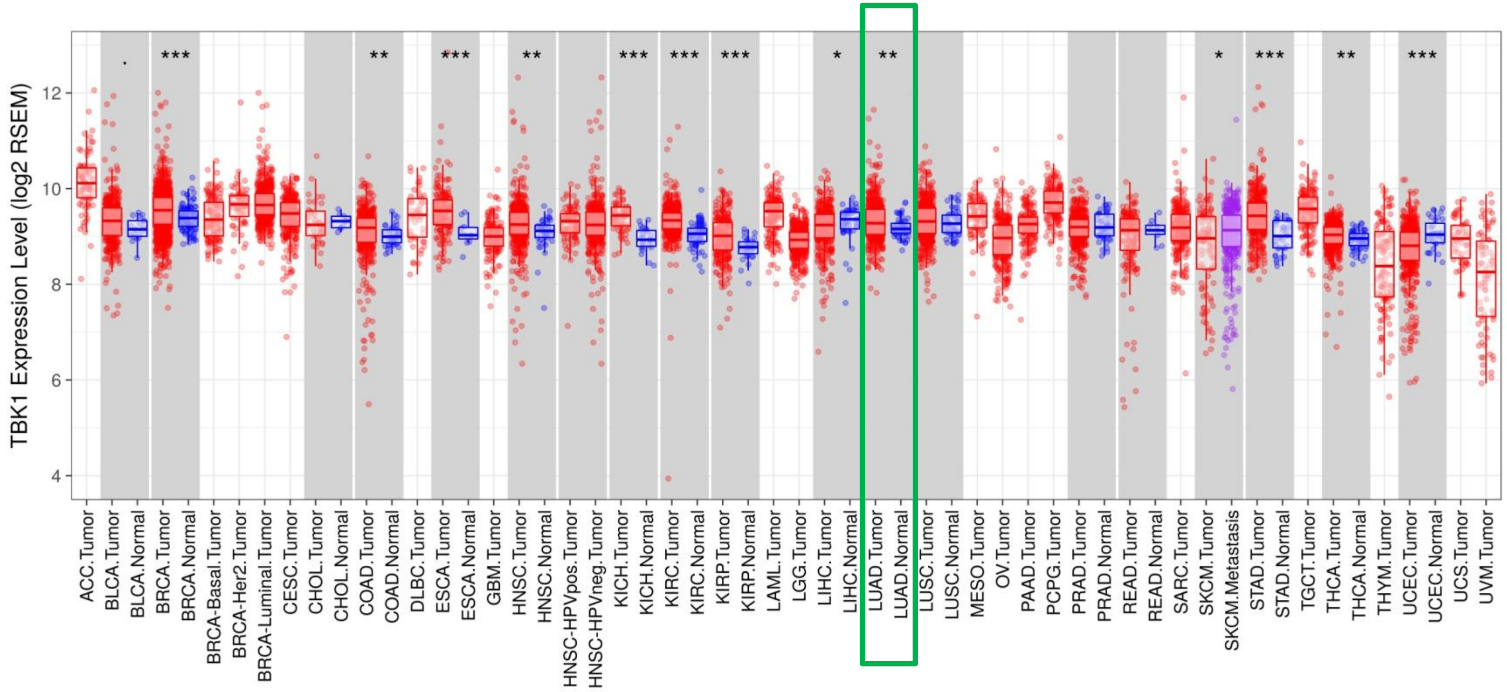
TBK1 Expression Level (log2 RSEM)
12
10
8
6
4
ACC.Tumor
BLCA.Tumor
BLCA.Normal
BRCA.Tumor
BRCA.Normal
BRCA-Basal.Tumor
BRCA-Her2.Tumor
BRCA-Luminal.Tumor
CESC.Tumor
CHOL.Tumor
CHOL.Normal
COAD.Tumor
COAD.Normal
DLBC.Tumor
ESCA.Tumor
ESCA.Normal
GBM.Tumor
HNSC.Tumor
HNSC.Normal
HNSC-HPVpos.Tumor
HNSC-HPVneg.Tumor
KICH.Tumor
KICH.Normal
KIRC.Tumor
KIRC.Normal
KIRP.Tumor
KIRP.Normal
LAML.Tumor
LGG.Tumor
LIHC.Tumor
LIHC.Normal
LUAD.Tumor
LUAD.Normal
LUSC.Tumor
LUSC.Normal
MESO.Tumor
OV.Tumor
PAAD.Tumor
PCPG.Tumor
PRAD.Tumor
PRAD.Normal
READ.Tumor
READ.Normal
SARC.Tumor
SKCM.Tumor
SKCM.Metastasis
STAD.Tumor
STAD.Normal
TGCT.Tumor
THCA.Tumor
THCA.Normal
THYM.Tumor
UCEC.Tumor
UCEC.Normal
UCS.Tumor
UVM.Tumor

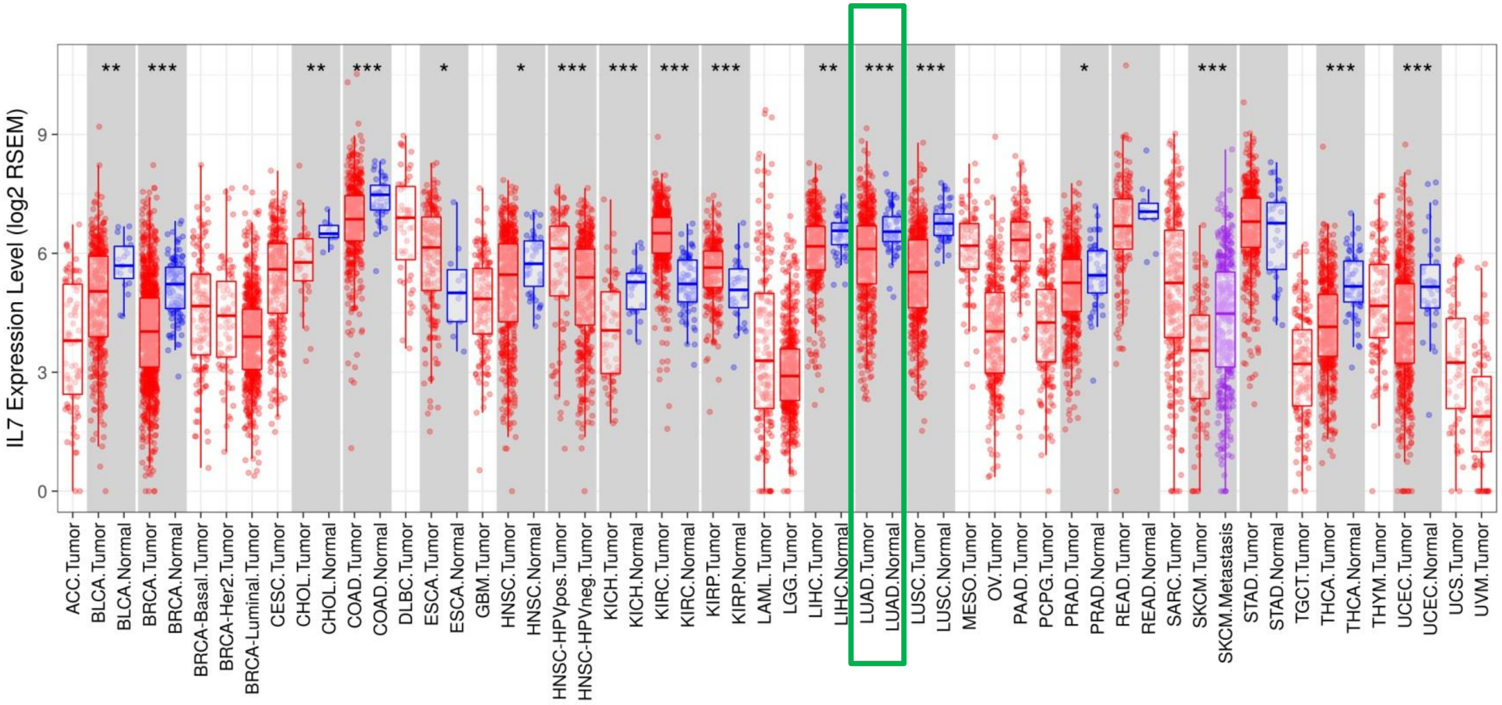
IL7 Expression Level (log2 RSEM)
0
3
6
9
**
***
**
***
*
*
***
***
***
***
**
***
***
*
***
***
***
ACC.Tumor
BLCA.Tumor
BLCA.Normal
BRCA.Tumor
BRCA.Normal
BRCA-Basal.Tumor
BRCA-Her2.Tumor
BRCA-Luminal.Tumor
CESC.Tumor
CHOL.Tumor
CHOL.Normal
COAD.Tumor
COAD.Normal
DLBC.Tumor
ESCA.Tumor
ESCA.Normal
GBM.Tumor
HNSC.Tumor
HNSC.Normal
HNSC-HPVpos.Tumor
HNSC-HPVneg.Tumor
KICH.Tumor
KICH.Normal
KIRC.Tumor
KIRC.Normal
KIRP.Tumor
KIRP.Normal
LAML.Tumor
LGG.Tumor
LIHC.Tumor
LIHC.Normal
LUAD.Tumor
LUAD.Normal
LUSC.Tumor
LUSC.Normal
MESO.Tumor
OV.Tumor
PAAD.Tumor
PCPG.Tumor
PRAD.Tumor
PRAD.Normal
READ.Tumor
READ.Normal
SARC.Tumor
SKCM.Tumor
SKCM.Metastasis
STAD.Tumor
STAD.Normal
TGCT.Tumor
THCA.Tumor
THCA.Normal
THYM.Tumor
UCEC.Tumor
UCEC.Normal
UCS.Tumor
UVM.Tumor

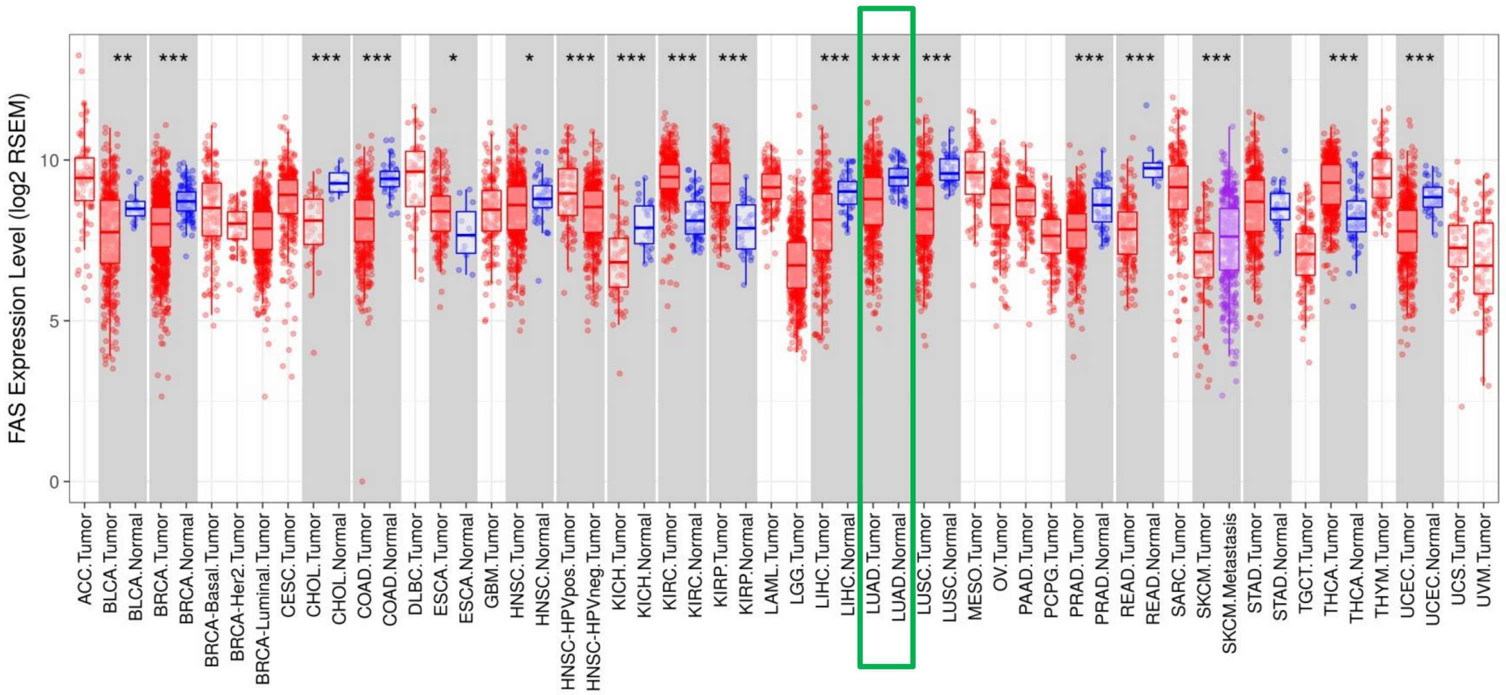
FAS Expression Level (log2 RSEM)
0
5
10
** *** *** *** * * *** *** *** *** *** *** *** *** *** *** *** ***
ACC.Tumor
BLCA.Tumor
BLCA.Normal
BRCA.Tumor
BRCA.Normal
BRCA-Basal.Tumor
BRCA-Her2.Tumor
BRCA-Luminal.Tumor
CESC.Tumor
CHOL.Tumor
CHOL.Normal
COAD.Tumor
COAD.Normal
DLBC.Tumor
ESCA.Tumor
ESCA.Normal
GBM.Tumor
HNSC.Tumor
HNSC.Normal
HNSC-HPVpos.Tumor
HNSC-HPVneg.Tumor
KICH.Tumor
KICH.Normal
KIRC.Tumor
KIRC.Normal
KIRP.Tumor
KIRP.Normal
LAML.Tumor
LGG.Tumor
LIHC.Tumor
LIHC.Normal
LUAD.Tumor
LUAD.Normal
LUSC.Tumor
LUSC.Normal
MESO.Tumor
OV.Tumor
PAAD.Tumor
PCPG.Tumor
PRAD.Tumor
PRAD.Normal
READ.Tumor
READ.Normal
SARC.Tumor
SKCM.Tumor
SKCM.Metastasis
STAD.Tumor
STAD.Normal
TGCT.Tumor
THCA.Tumor
THCA.Normal
THYM.Tumor
UCEC.Tumor
UCEC.Normal
UCS.Tumor
UVM.Tumor

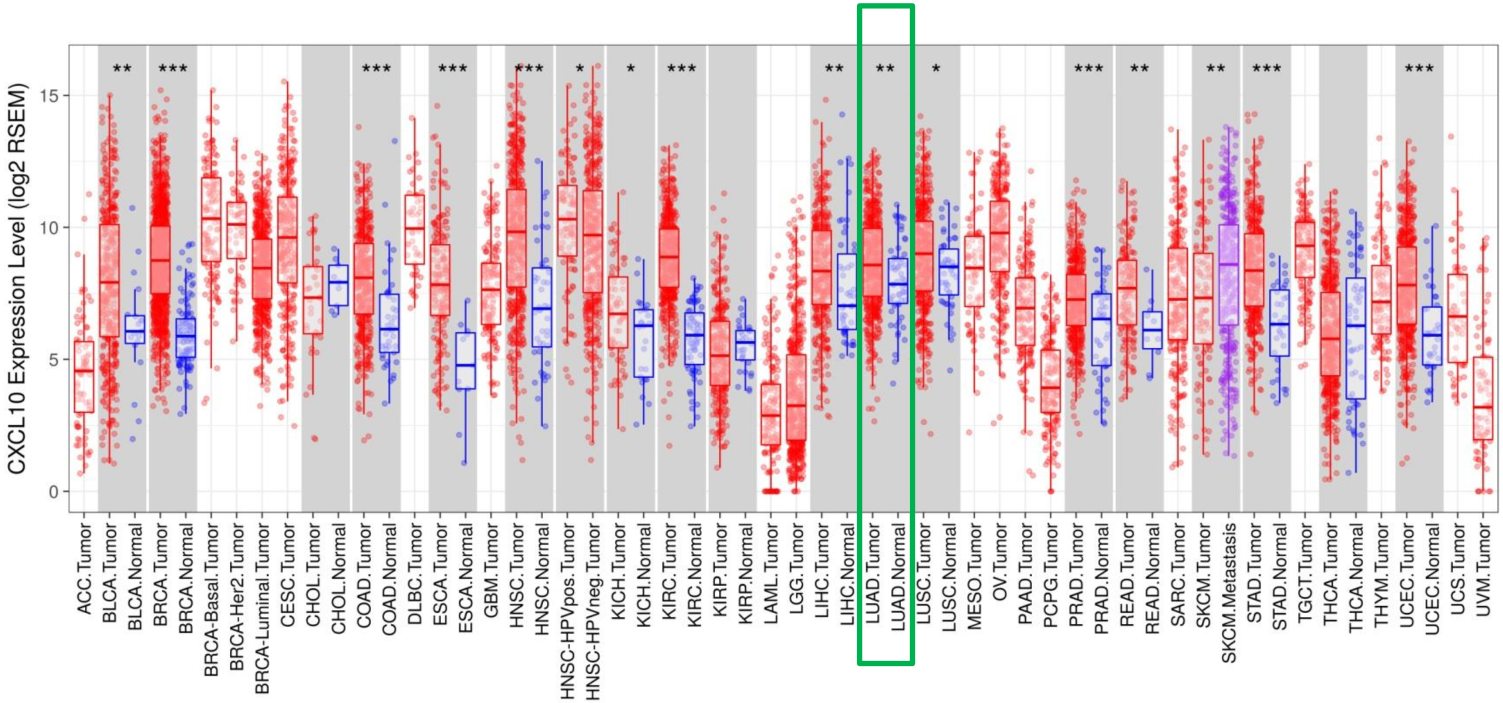
CXCL10 Expression Level (log2 RSEM)
0
5
10
15
** *** *** *** *** * * *** ** ** * *** ** ** *** ***
ACC.Tumor
BLCA.Tumor
BLCA.Normal
BRCA.Tumor
BRCA.Normal
BRCA-Basal.Tumor
BRCA-Her2.Tumor
BRCA-Luminal.Tumor
CESC.Tumor
CHOL.Tumor
CHOL.Normal
COAD.Tumor
COAD.Normal
DLBC.Tumor
ESCA.Tumor
ESCA.Normal
GBM.Tumor
HNSC.Tumor
HNSC.Normal
HNSC-HPVpos.Tumor
HNSC-HPVneg.Tumor
KICH.Tumor
KICH.Normal
KIRC.Tumor
KIRC.Normal
KIRP.Tumor
KIRP.Normal
LAML.Tumor
LGG.Tumor
LIHC.Tumor
LIHC.Normal
LUAD.Tumor
LUAD.Normal
LUSC.Tumor
LUSC.Normal
MESO.Tumor
OV.Tumor
PAAD.Tumor
PCPG.Tumor
PRAD.Tumor
PRAD.Normal
READ.Tumor
READ.Normal
SARC.Tumor
SKCM.Tumor
SKCM.Metastasis
STAD.Tumor
STAD.Normal
TGCT.Tumor
THCA.Tumor
THCA.Normal
THYM.Tumor
UCEC.Tumor
UCEC.Normal
UCS.Tumor
UVM.Tumor

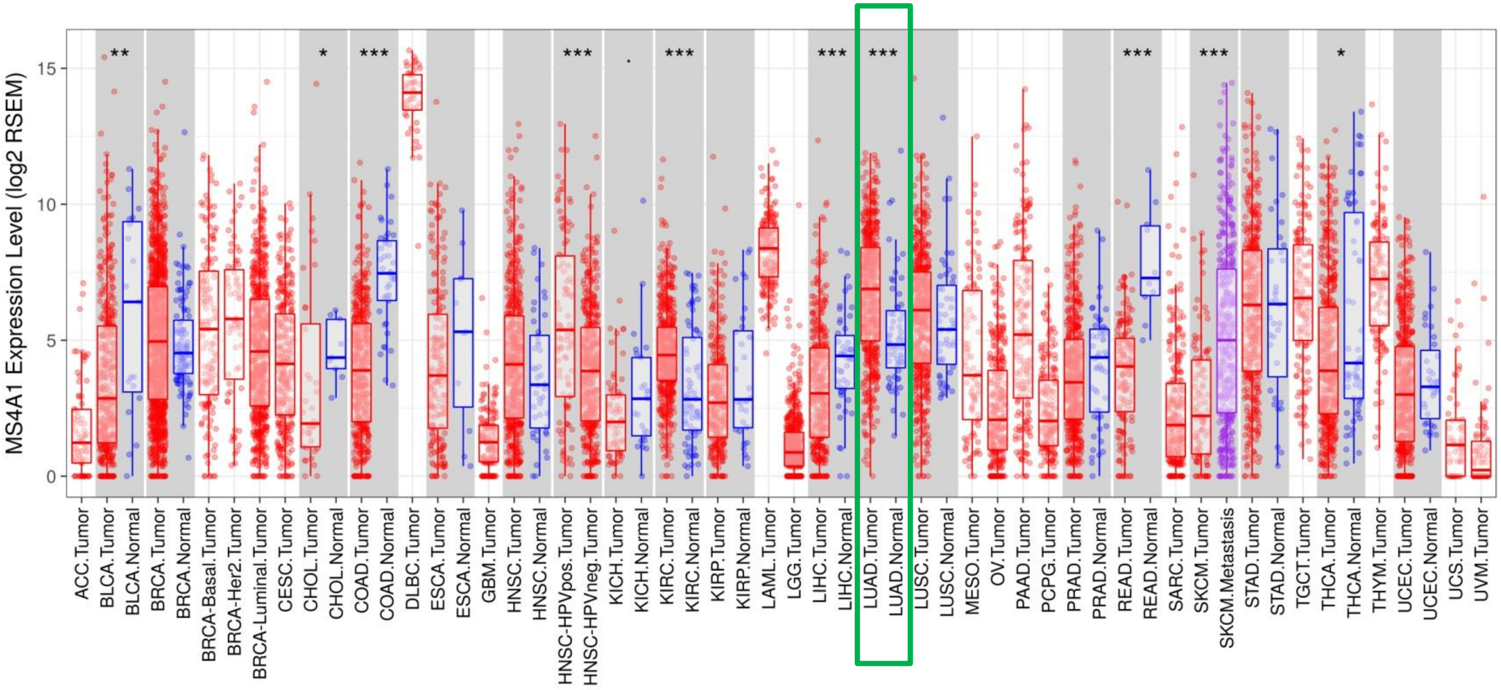

MS4A1 Expression Level (log2 RSEM)
15
10
5
0
**
*
***
***
.
***
***
***
***
***
*
ACC.Tumor
BLCA.Tumor
BLCA.Normal
BRCA.Tumor
BRCA.Normal
BRCA-Basal.Tumor
BRCA-Her2.Tumor
BRCA-Luminal.Tumor
CESC.Tumor
CHOL.Tumor
CHOL.Normal
COAD.Tumor
COAD.Normal
DLBC.Tumor
ESCA.Tumor
ESCA.Normal
GBM.Tumor
HNSC.Tumor
HNSC.Normal
HNSC-HPVpos.Tumor
HNSC-HPVneg.Tumor
KICH.Tumor
KICH.Normal
KIRC.Tumor
KIRC.Normal
KIRP.Tumor
KIRP.Normal
LAML.Tumor
LGG.Tumor
LIHC.Tumor
LIHC.Normal
LUAD.Tumor
LUAD.Normal
LUSC.Tumor
LUSC.Normal
MESO.Tumor
OV.Tumor
PAAD.Tumor
PCPG.Tumor
PRAD.Tumor
PRAD.Normal
READ.Tumor
READ.Normal
SARC.Tumor
SKCM.Tumor
SKCM.Metastasis
STAD.Tumor
STAD.Normal
TGCT.Tumor
THCA.Tumor
THCA.Normal
THYM.Tumor
UCEC.Tumor
UCEC.Normal
UCS.Tumor
UVM.Tumor

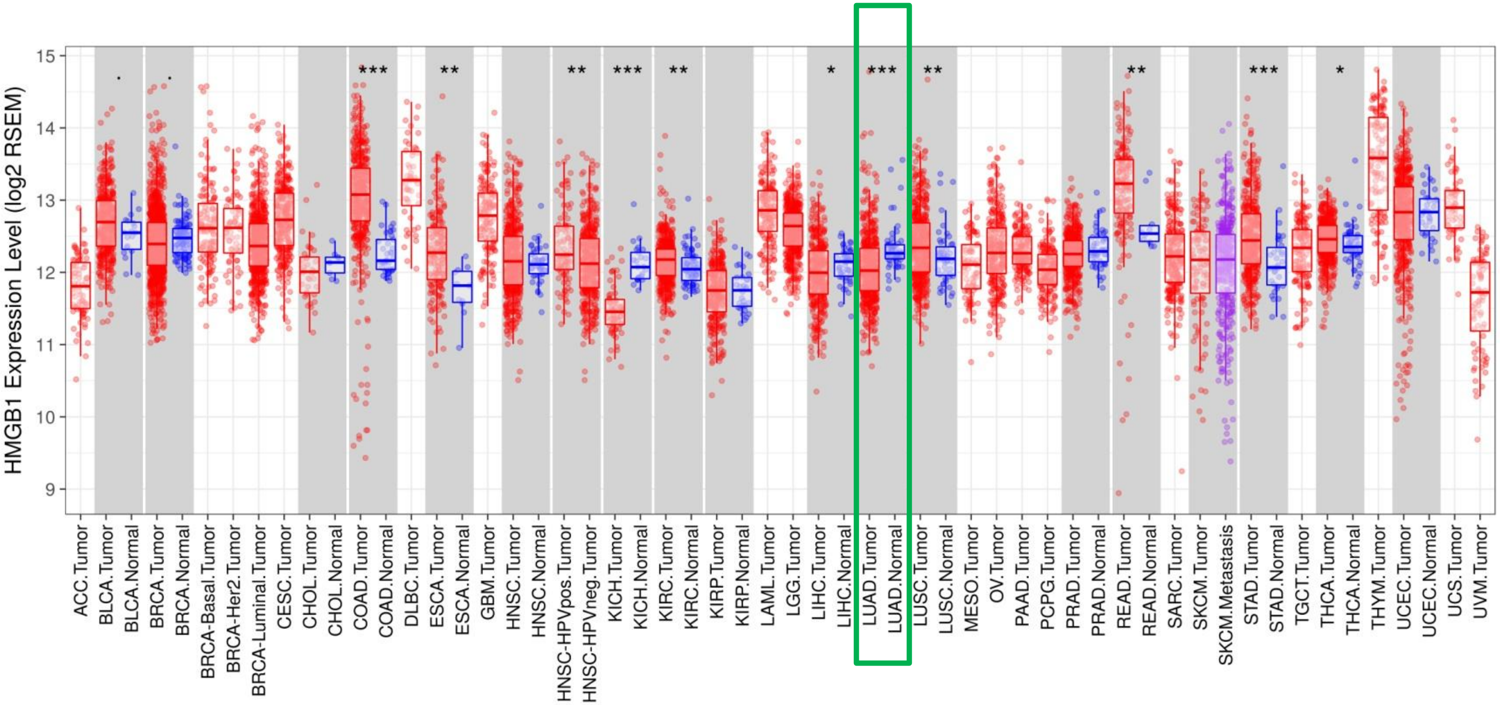
HMGB1 Expression Level (log2 RSEM)
ACC.Tumor
BLCA.Tumor
BLCA.Normal
BRCA.Tumor
BRCA.Normal
BRCA-Basal.Tumor
BRCA-Her2.Tumor
BRCA-Luminal.Tumor
CESC.Tumor
CHOL.Tumor
CHOL.Normal
COAD.Tumor
COAD.Normal
DLBC.Tumor
ESCA.Tumor
ESCA.Normal
GBM.Tumor
HNSC.Tumor
HNSC.Normal
HNSC-HPVpos.Tumor
HNSC-HPVneg.Tumor
KICH.Tumor
KICH.Normal
KIRC.Tumor
KIRC.Normal
KIRP.Tumor
KIRP.Normal
LAML.Tumor
LGG.Tumor
LIHC.Tumor
LIHC.Normal
LUAD.Tumor
LUAD.Normal
LUSC.Tumor
LUSC.Normal
MESO.Tumor
OV.Tumor
PAAD.Tumor
PCPG.Tumor
PRAD.Tumor
PRAD.Normal
READ.Tumor
READ.Normal
SARC.Tumor
SKCM.Tumor
SKCM.Metastasis
STAD.Tumor
STAD.Normal
TGCT.Tumor
THCA.Tumor
THCA.Normal
THYM.Tumor
UCEC.Tumor
UCEC.Normal
UCS.Tumor
UVM.Tumor

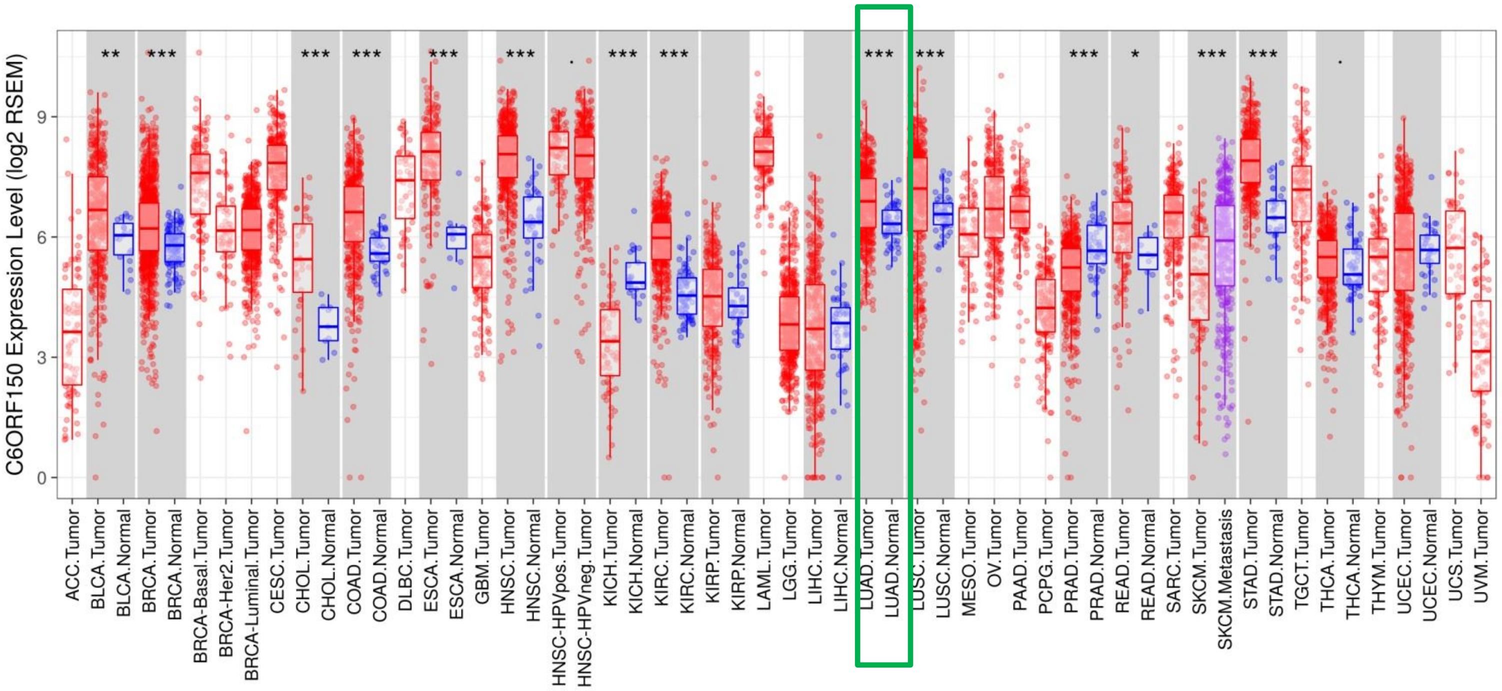
C6ORF150 Expression Level (log2 RSEM)
0
3
6
9
**
***
***
***
***
***
.
***
***
***
***
***
***
*
***
***
.
ACC.Tumor
BLCA.Tumor
BLCA.Normal
BRCA.Tumor
BRCA.Normal
BRCA-Basal.Tumor
BRCA-Her2.Tumor
BRCA-Luminal.Tumor
CESC.Tumor
CHOL.Tumor
CHOL.Normal
COAD.Tumor
COAD.Normal
DLBC.Tumor
ESCA.Tumor
ESCA.Normal
GBM.Tumor
HNSC.Tumor
HNSC.Normal
HNSC-HPVpos.Tumor
HNSC-HPVneg.Tumor
KICH.Tumor
KICH.Normal
KIRC.Tumor
KIRC.Normal
KIRP.Tumor
KIRP.Normal
LAML.Tumor
LGG.Tumor
LIHC.Tumor
LIHC.Normal
LUAD.Tumor
LUAD.Normal
LUSC.Tumor
LUSC.Normal
MESO.Tumor
OV.Tumor
PAAD.Tumor
PCPG.Tumor
PRAD.Tumor
PRAD.Normal
READ.Tumor
READ.Normal
SARC.Tumor
SKCM.Tumor
SKCM.Metastasis
STAD.Tumor
STAD.Normal
TGCT.Tumor
THCA.Tumor
THCA.Normal
THYM.Tumor
UCEC.Tumor
UCEC.Normal
UCS.Tumor
UVM.Tumor

*MB21D1

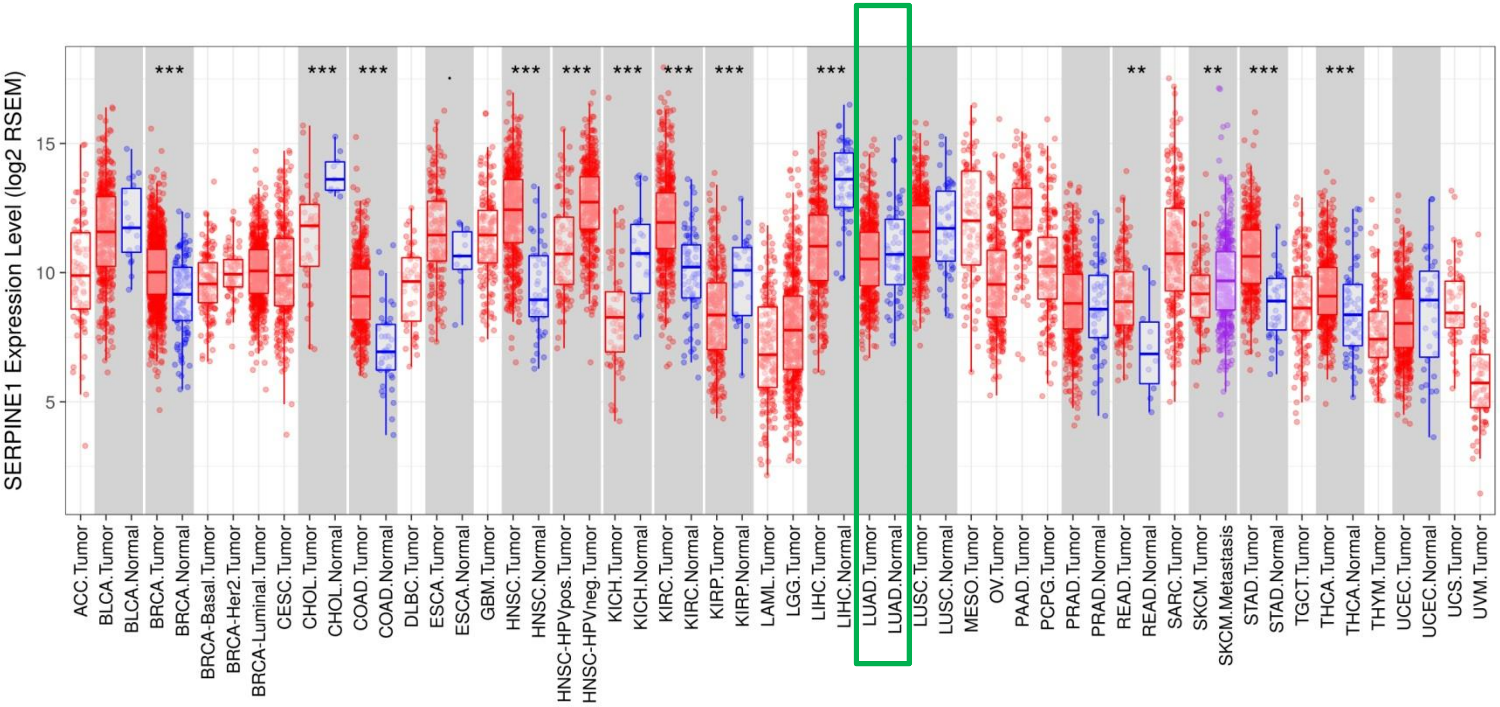

SERPINE1 Expression Level (log2 RSEM)
15
10
5
***
***
***
.
***
***
***
***
***
***
**
**
***
***
ACC.Tumor
BLCA.Tumor
BLCA.Normal
BRCA.Tumor
BRCA.Normal
BRCA-Basal.Tumor
BRCA-Her2.Tumor
BRCA-Luminal.Tumor
CESC.Tumor
CHOL.Tumor
CHOL.Normal
COAD.Tumor
COAD.Normal
DLBC.Tumor
ESCA.Tumor
ESCA.Normal
GBM.Tumor
HNSC.Tumor
HNSC.Normal
HNSC-HPVpos.Tumor
HNSC-HPVneg.Tumor
KICH.Tumor
KICH.Normal
KIRC.Tumor
KIRC.Normal
KIRP.Tumor
KIRP.Normal
LAML.Tumor
LGG.Tumor
LIHC.Tumor
LIHC.Normal
LUAD.Tumor
LUAD.Normal
LUSC.Tumor
LUSC.Normal
MESO.Tumor
OV.Tumor
PAAD.Tumor
PCPG.Tumor
PRAD.Tumor
PRAD.Normal
READ.Tumor
READ.Normal
SARC.Tumor
SKCM.Tumor
SKCM.Metastasis
STAD.Tumor
STAD.Normal
TGCT.Tumor
THCA.Tumor
THCA.Normal
THYM.Tumor
UCEC.Tumor
UCEC.Normal
UCS.Tumor
UVM.Tumor

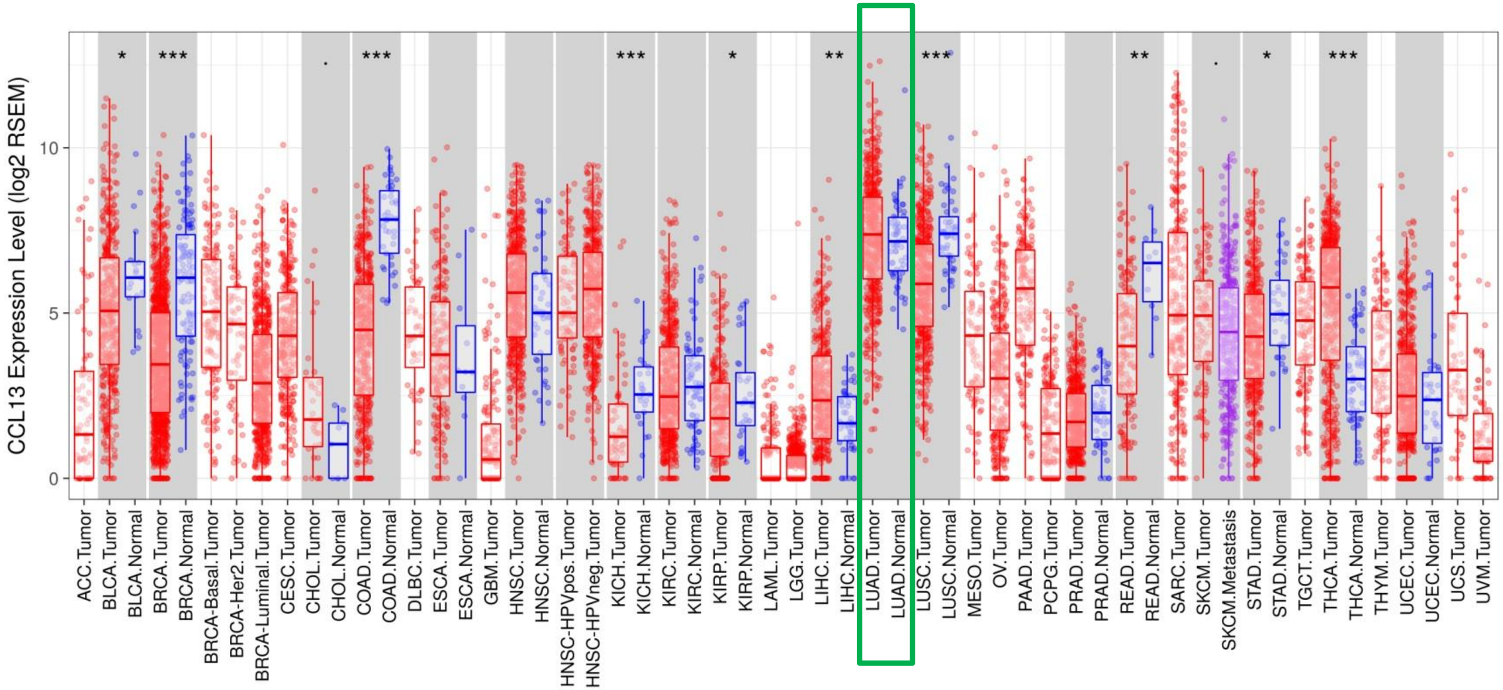
CCL13 Expression Level (log2 RSEM)
0
5
10
*
***
.
***
***
*
**
***
**
.
*
***
ACC.Tumor
BLCA.Tumor
BLCA.Normal
BRCA.Tumor
BRCA.Normal
BRCA-Basal.Tumor
BRCA-Her2.Tumor
BRCA-Luminal.Tumor
CESC.Tumor
CHOL.Tumor
CHOL.Normal
COAD.Tumor
COAD.Normal
DLBC.Tumor
ESCA.Tumor
ESCA.Normal
GBM.Tumor
HNSC.Tumor
HNSC.Normal
HNSC-HPVpos.Tumor
HNSC-HPVneg.Tumor
KICH.Tumor
KICH.Normal
KIRC.Tumor
KIRC.Normal
KIRP.Tumor
KIRP.Normal
LAML.Tumor
LGG.Tumor
LIHC.Tumor
LIHC.Normal
LUAD.Tumor
LUAD.Normal
LUSC.Tumor
LUSC.Normal
MESO.Tumor
OV.Tumor
PAAD.Tumor
PCPG.Tumor
PRAD.Tumor
PRAD.Normal
READ.Tumor
READ.Normal
SARC.Tumor
SKCM.Tumor
SKCM.Metastasis
STAD.Tumor
STAD.Normal
TGCT.Tumor
THCA.Tumor
THCA.Normal
THYM.Tumor
UCEC.Tumor
UCEC.Normal
UCS.Tumor
UVM.Tumor
